# TH301 Emerges as a Novel Anti-Oncogenic Agent for Human Pancreatic Cancer Cells: The Dispensable Roles of p53, CRY2 and BMAL1 in TH301-Induced *CDKN1A*/p21^CIP1/WAF1^ Upregulation

**DOI:** 10.3390/ijms26010178

**Published:** 2024-12-28

**Authors:** Danae Farmakis, Dimitrios J. Stravopodis, Anastasia Prombona

**Affiliations:** 1 Section of Cell Biology and Biophysics, Department of Biology, School of Science, National and Kapodistrian University of Athens (NKUA), Panepistimiopolis, Zografou, 157 01 Athens, Greece; danaefar@bio.demokritos.gr; 2 Laboratory of Chronobiology, Institute of Biosciences and Applications (IBA), National Centre for Scientific Research (NCSR) “Demokritos”, 153 41 Aghia Paraskevi, Greece; prombona@bio.demokritos.gr

**Keywords:** apoptosis, autophagy, BMAL1, *CDKN1A*, circadian clock, CRY2, PDAC, p21^CIP1/WAF1^, p53, TH301

## Abstract

*Background*: Pancreatic Ductal Adeno-Carcinoma (PDAC) is a highly aggressive cancer, with limited treatment options. Disruption of the circadian clock, which regulates key cellular processes, has been implicated in PDAC initiation and progression. Hence, targeting circadian clock components may offer new therapeutic opportunities for the disease. This study investigates the cytopathic effects of TH301, a novel CRY2 stabilizer, on PDAC cells, aiming to evaluate its potential as a novel therapeutic agent. *Methods*: PDAC cell lines (AsPC-1, BxPC-3 and PANC-1) were treated with TH301, and cell viability, cell cycle progression, apoptosis, autophagy, circadian gene, and protein expression profiles were analyzed, using MTT assay, flow cytometry, Western blotting, and RT-qPCR technologies. *Results*: TH301 proved to significantly decrease cell viability and to induce cell cycle arrest at the G1-phase across all PDAC cell lines herein examined, especially the AsPC-1 and BxPC-3 ones. It caused dose-dependent apoptosis and autophagy, and it synergized with Chloroquine and Oxaliplatin to enhance anti-oncogenicity. The remarkable induction of p21 by TH301 was shown to follow clock- and p53-independent patterns, thereby indicating the critical engagement of alternative mechanisms. *Conclusions*: TH301 demonstrates significant anti-cancer activities in PDAC cells, thus serving as a promising new therapeutic agent, which can also synergize with approved treatment schemes by targeting pathways beyond circadian clock regulation. Altogether, TH301 likely opens new therapeutic windows for the successful management of pancreatic cancer in clinical practice.

## 1. Introduction

Pancreatic Ductal Adeno-Carcinoma (PDAC) is one of the most aggressive forms of cancer, characterized by a high mortality rate and poor prognosis. Globally, PDAC is the twelfth most common malignancy, with an estimated 510,566 new cases in 2022, and the sixth leading cause of cancer mortality, accounting for 467,005 deaths worldwide [1]. Despite advances in cancer research and treatment, the 5-year survival rate for PDAC remains dismal at less than 13%, thus rendering PDAC challenging to diagnose early and treat effectively [2].

Current treatment modalities for PDAC include chemotherapy, radiation, and surgery. Radiotherapy and common chemotherapy treatments used in the management of PDAC include Gemcitabine, nab-paclitaxel, and combination regimens, such as FOLFIRINOX (Folinic Acid, 5-Fluorouracil, Irinotecan and Oxaliplatin). Notably, FOLFIRINOX has been shown to act more effectively than Gemcitabine in improving survival outcomes, particularly in patients with good performance status [3]. Despite their broad clinical applications, these treatments pose significant challenges and have limitations. In addition to treatment exhibiting high toxicity and serious side effects, the major difficulties in managing PDAC stem from inherent or acquired resistance mechanisms, such as activation of compensatory pathways, and aberrations in drug transport and metabolism processes. Development of resistance to chemotherapy and radiotherapy seems to be significantly influenced by genetic lesions in specific genes, such as the *KRAS*, *CDKN2A*, *TP53*, and *SMAD4* ones, along with the simultaneous activation of downstream signaling pathways [4,5]. Recent efforts focusing on functional inhibition of the mutated KRAS protein in PDAC have shown promising pre-clinical and clinical results [6]. However, cancer cells often develop compensatory escape mechanisms to counteract the efficacy of KRAS inhibitors [7]. Most importantly, the complex microenvironment of the tumors and presence of cancer stem cells (CSCs) further complicate, and frequently compromise, treatment efforts [8,9].

These challenges necessitate the prompt development of alternative therapeutic strategies. One emerging area of interest is the pharmacological modulation of the cell circadian clock [10]. The circadian clock is an intrinsic time-keeping system that regulates gene expression and various biological functions across all kingdoms of life [11]. At the molecular level, it consists of transcription–translation feedback loops that maintain rhythmic expression of genes involved in critical cellular processes, like metabolism, redox regulation, autophagy, DNA repair, and cell cycle control [12,13,14,15]. The “positive arm” of this feedback loop includes BMAL1 (Brain and Muscle ARNT-Like 1) and CLOCK (Circadian Locomotor Output Cycles Kaput) proteins, which form a complex that activates E-box-mediated transcription of target genes, including *Period* (*PER1*, *2* and *3*) and *Cryptochrome* (*CRY1* and *2*), which in turn constitute the “negative arm”. PER and CRY proteins accumulate in the cytoplasm, form heterodimers, and translocate into the nucleus to inhibit the BMAL1 complex, thereby repressing their own transcription [16]. This feedback inhibition creates a roughly 24 h cycle of gene expression. Additional feedback loops involving nuclear receptors, like REV-ERBs and RORs, further stabilize the clock by regulating *BMAL1* transcription via its promoter ROR elements [17,18]. Cell cycle key genes, essentially controlling the G1/S and G2/M transitions, are also transcriptionally activated by the BMAL1 complex, since their respective promoters contain E-boxes [19]. Likewise, Gréchez-Cassiau and collaborators have previously reported in hepatocytes that clock genes can control the G1/S progression through regulation of the *CDKN1A* gene, which encodes for the p21^CIP1/WAF1^ (p21) protein, a major Cyclin/CDK inhibitor, with alterations of the circadian clock leading to changes in p21 expression and aberrations in cell proliferation [20].

Deregulation of circadian clock plays crucial roles in tumorigenesis by promoting rapid proliferation, increased metabolic demand, and resistance to apoptosis [21,22]. In PDAC, disruption of circadian clock can be evidenced by bioinformatics and gene expression studies [23], revealing altered activities of core clock genes, as compared to normal pancreatic tissue-derived ones, while it is associated with faster tumor growth, reduced survival rate, and increased chemotherapy resistance [24]. Importantly, small molecules that modulate circadian clock components have shown promise in pre-clinical studies by disrupting cancer cell proliferation and enhancing existing treatment efficacies [10]. CRY stabilizers, such as the KL001 compound and its derivatives, have been shown to extend survival in glioblastoma models by downregulating stemness genes and inducing apoptosis [25,26]. On the other hand, CRY inhibitors, like KS15, have demonstrated effectiveness in breast cancer cells by disrupting CRY-mediated transcriptional repression, leading to cell cycle arrest, apoptosis, and increased sensitivity to chemotherapy [27,28]. Other clock modulators, including REV-ERB and ROR agonists, have also presented promising anti-cancer activities, further supporting the potential of targeting circadian clock components as a novel therapeutic strategy [29,30,31,32,33].

Taken together, PDAC remains a major challenge in medical and molecular oncology, immediately requiring innovative treatment approaches. The cardinal circadian clock’s role in cell cycle regulation, as well as cancer initiation and progression, renders the circadian clock a promising therapeutic target. Hence, employment of circadian clock modulators is expected to improve current therapies or offer new options able to clinically benefit PDAC-affected patients. Towards this direction, we have focused herein on the pivotal clock component CRY2, which is critically implicated in DNA repair, cell cycle regulation, and chemoresistance modulation, thereby rendering CRY2 a novel and important (druggable) target for pancreatic cancer therapy [23,24]. Given that the synthetic chemical compound TH301 {1-(4-Chlorophenyl)-N-[2,6-Dihydro-2-(4-Methoxyphenyl)-5,5-Dioxido-4H-Thieno [3,4-c]Pyrazol-3-yl]-Cyclopentanecarboxamide} acts as a bona fide—novel—CRY2 stabilizer [33,34], by mechanistically investigating the TH301-induced sub-routines that can cause severe PDAC pathologies, we aim to introduce new strategies, with strong promise for more effective treatments and better outcomes for the disease.

## 2. Results

### 2.1. TH301-Induced Reduction of Cell Viability and Growth of Pancreatic Cancer Cells in a Mutational Signature-Dependent Fashion

**Figure 1:**
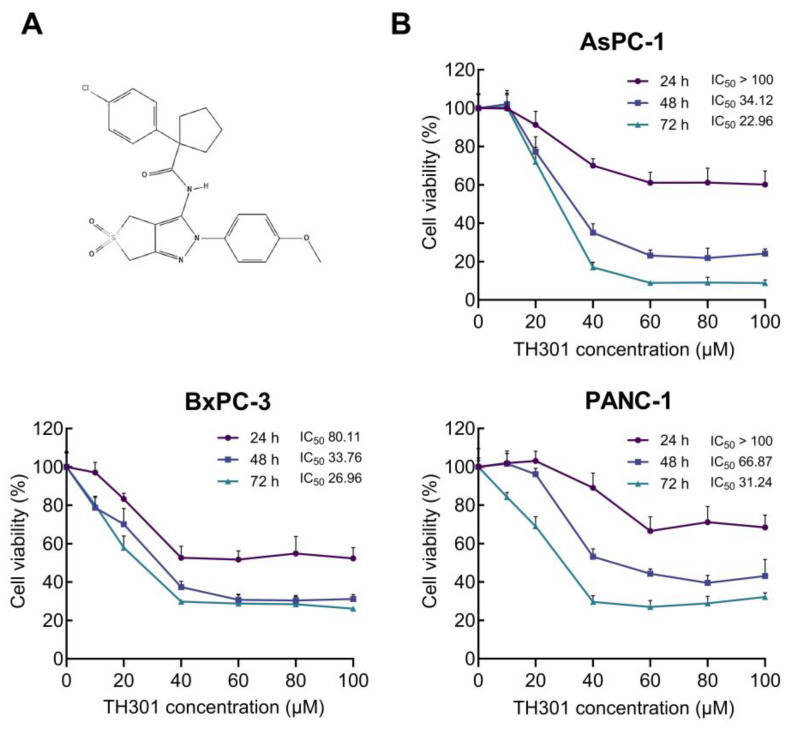
TH301 reduces human pancreatic cancer cell viability in a dose- and time-dependent manner. (**A**) Chemical structure of TH301 {1-(4-Chlorophenyl)-N-[2,6-Dihydro-2-(4-Methoxyphenyl)-5,5-Dioxido-4H-Thieno [3,4-c]Pyrazol-3-yl]-Cyclopentanecarboxamide} (https://pubchem.ncbi.nlm.nih.gov/compound/4653191) [34]. (**B**) Cell viability (%) graphs of AsPC-1, BxPC-3, and PANC-1 cells after treatment with increasing concentrations (0, 20, 40, 60, 80, and 100 μM) of TH301, for 24, 48, and 72 h (post-administration). Data from 6 replicates are presented as mean ± SD values. IC_50_ values were measured through employment of a non-linear regression model.

To demonstrate that circadian clock proteins can indeed be modulated in PDAC, we first analyzed the mutational frequencies of core circadian clock genes compared to key PDAC mutations in a cohort of 185 PDAC patients (Supplementary Figure S1). This analysis highlighted the low probability of mutations in core clock genes, thus indicating that circadian clock functionality can be effectively targeted with specific modulators in PDAC settings. Hence, to explore the effects of TH301 (Figure 1A), a bona fide CRY2 stabilizer, on PDAC “modelled” pathology, herein we examined the human pancreatic cancer cell lines AsPC-1, BxPC-3, and PANC-1, with each one of them characterized by a distinct mutational signature detailed in Table S2 (https://depmap.org; accessed on 20 November 2024). To determine the pathogenic responses of TH301 regarding cell viability and growth, the three PDAC cell lines were treated with increasing concentrations (0–100 μΜ) of TH301, for 24, 48, and 72 h. Cell survival was assessed and quantified by MTT assays. As shown in Figure 1B, TH301 administration resulted in a dose- and time-dependent significant decrease in the viability of all three cell lines examined. Both AsPC-1 and BxPC-3 cell lines presented a notable reduction in cell viability after 48 h of treatment with 40 μM TH301. In contrast, PANC-1 cells showed comparatively lower sensitivity, requiring 72 h, or 60 μM, to achieve a similar decrease rate of cell viability (~60% reduction). Most importantly, AsPC-1 cells were presented with severe pathologies after their exposure to TH301 for 72 h, having the observed viabilities almost eliminated at the 60, 80, and 100 μM of TH301 concentration (Figure 1B). Of note, TH301 concentrations lower than 1 μM did not exhibit any detectable effect on cell viability (based on MTT assays), whereas doses exceeding 100 μM encountered severe solvent (DMSO) effects.

These results prove that TH301 can cause strong cytotoxic effects in human pancreatic cancer cells, following dose-, time-, and cell type-dependent patterns. In the same direction, we next performed MTT assays to evaluate and quantify the pathogenic effects of KL001, a CRY1/2 stabilizer [25,26], and KS15, a CRY1/2 inhibitor [27,28], on AsPC-1, BxPC-3, and PANC-1 cell viabilities. Treatment with KL001 was less effective than TH301 in reducing cell viability, whereas KS15 showed absence of significant reduction in cell viabilities (Supplementary Figure S2). However, AsPC-1 cells were characterized by a prominent decline in their viability and growth at the highest dose (i.e., 100 μM) and longest time (i.e., 72 h) of each agent (i.e., KL001 or KS15) administration. Taken together, the CRY stabilizer, TH301, demonstrates a superior efficacy in reducing human pancreatic cancer cell viability and growth, compared to other CRY modulators tested herein (KL001 and KS15).

### 2.2. TH301 Causes Cell Cycle Arrest at the G1-Phase and Alters Protein Expression Profiles of Critical Regulators in Pancreatic Cancer Cells: Mutational Load-Dependent Responses

**Figure 2:**
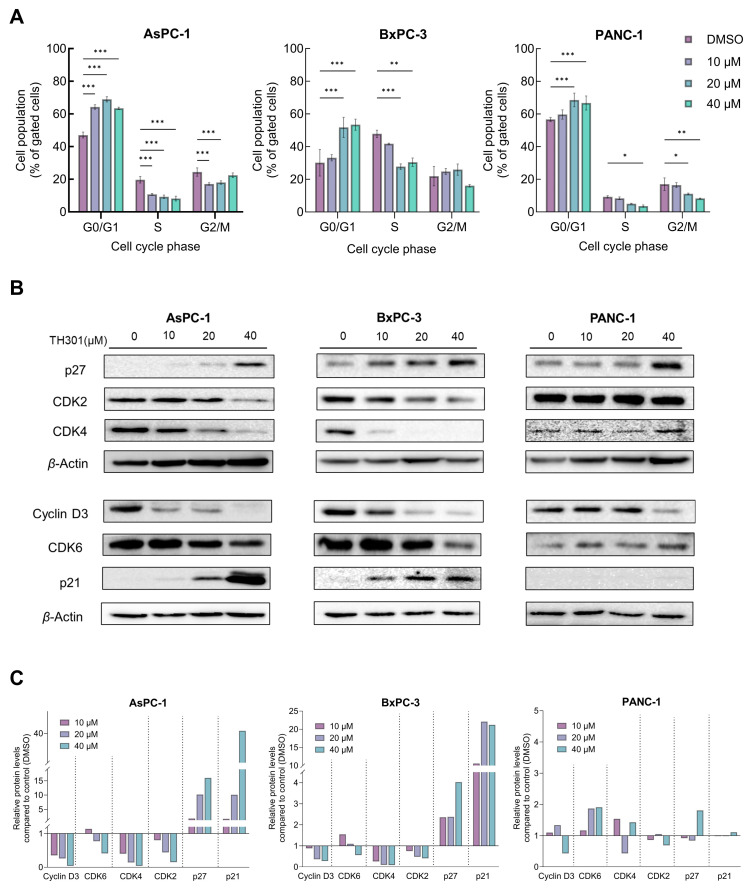
TH301 induces cell cycle arrest at the G1-phase and causes major expression alterations in cell cycle control proteins. (**A**) Flow cytometry (FACS) analysis of PI-stained PDAC cells, after treatment with increasing concentrations (0, 10, 20, and 40 μM) of TH301 for 24 h (post-administration). Data from 3 replicates (N = 3) are presented as mean ± SD values. Statistical significance was defined with one-way ANOVA, and comparisons were made in between control (0.1% DMSO) and (TH301) treated cells (% of control). Asterisks indicate comparisons between control and treated cells, at significance levels of 0.05 and below (*: <0.05; **: <0.01; ***: <0.001). (**B**) Western blotting-mediated expression profiling of main G1-phase-specific cell cycle regulators (Cyclin D3, CDK6, CDK4, CDK2, p21, and p27), after treatment of PDAC cells with increasing concentrations (0, 10, 20, and 40 μM) of TH301 for 24 h (post-administration). *β*-Actin was used as loading control (reference) protein. (**C**) Quantification of protein expression, as it is normalized to *β*-Actin (protein of reference), of G1-specific, cell cycle-phase proteins (Cyclin D3, CDK6, CDK4, CDK2, p21, and p27), of human pancreatic cancer cells, as compared to control cells (control cell values were set to “1”).

To investigate the underlying mechanism(s) of TH301-induced cell growth inhibition and survival compromise of PDAC cells, we first analyzed cell cycle phases after incubation of the cells with low to moderate TH301 doses (0–40 μΜ), for 24 h. As presented in Figure 2A, TH301 caused a dose-dependent arrest of cells at the G1-phase of the cell cycle. The increase in proportion of cells at the G1-phase was accompanied by a corresponding decrease in the proportion of cells at the S-phase. Notably, in PANC-1 cells, there was also a significant reduction observed in the percentage of cells at G2 following treatment with 40 µM TH301. Although this reduction was not statistically significant in BxPC-3 cells, there was a marked trend toward a decrease at the G2-phase cell percentage as well. To independently validate the results obtained from flow cytometry (FACS) analysis, we next examined the expression levels of proteins that critically control cell cycle progression, with major determinants being the Cyclin D3, CDK6 (kinase), CDK4 (kinase), CDK2 (kinase), and the CDK inhibitors p21^CIP1/WAF1^ (p21) and p27^KIP1^ (p27). After 24 h incubation with TH301, protein levels of cell cycle activators (i.e., Cyclin D3, CDK2, CDK4, and CDK6) decreased, whereas expression levels of CDK inhibitors (p21 and p27) increased significantly, in a dose-dependent manner for all three cell lines, with the exception of p21 protein in PANC-1 cells, which remained undetected (Figure 2B,C). These findings strongly suggest that TH301 can induce G1-arrest by severely altering the expression patterns of cell cycle regulatory proteins (i.e., p21 induction in AsPC-1 cells {40 μM TH301}; Figure 2B,C) in all three pancreatic cancer cell lines, albeit following mutational signature-specific profiles. In addition to cell cycle regulators, we also investigated the expression of genes coding for the major stemness factors *SOX2*, *NANOG*, and *OCT4*. Expression of stemness genes, particularly *NANOG*, was significantly reduced after pancreatic cancer cell exposure to TH301 (40 μM; 24 h) (Supplementary Figure S3). Altogether, in a pancreatic cancer cell environment, TH301 is able to induce cell cycle arrest at the G1-phase, and, simultaneously, attenuate stemness network activity via its transcription factor-program downregulation, thus highlighting the potential and promise of TH301 to act as a novel anti-proliferative and anti-survival agent for pancreatic cancer management and therapy.

### 2.3. TH301 Causes a Strong p53-Independent Induction of the CDKN1A/p21 Cell Cycle Inhibitor in Pancreatic Cancer Cell Environments, Following Mutational Load-Specific Patterns

**Figure 3:**
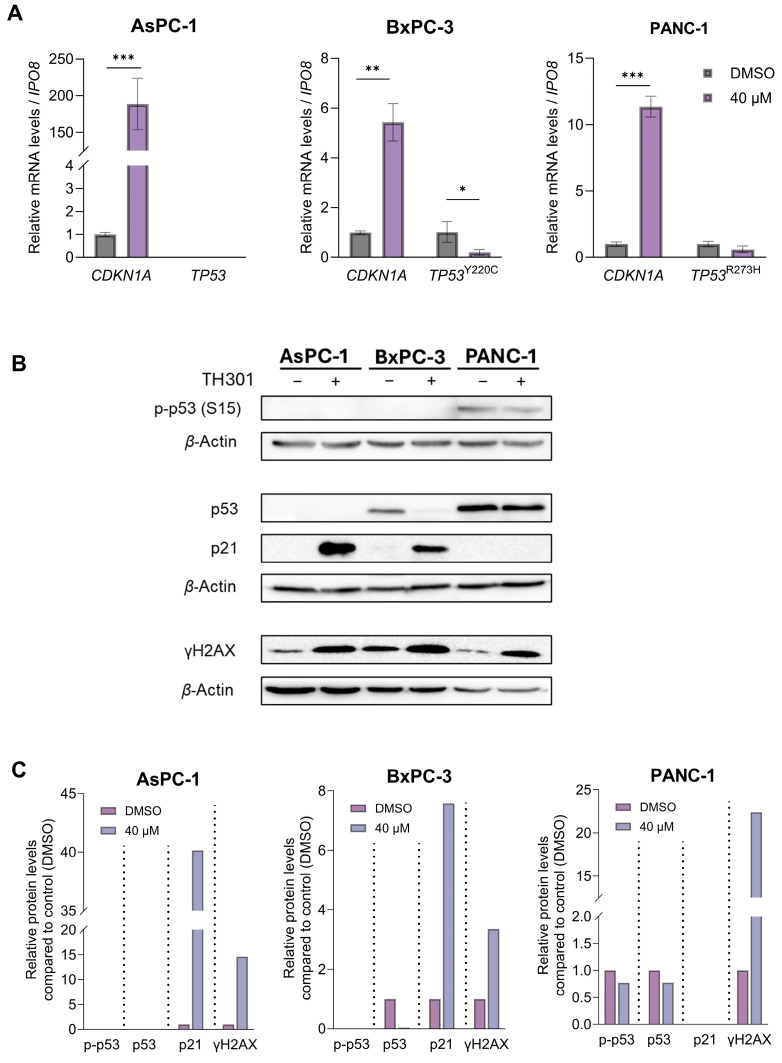
TH301-induced upregulation of *CDKN1A*/p21 follows p53-independent patterns. (**A**) mRNA levels of *CDKN1A* gene in AsPC-1, BxPC-3, and PANC-1 cells, and of mutant *TP53* gene in BxPC-3 and PANC-1 cells, following treatment with 40 μΜ TH301 for 24 h, were examined by RT-qPCR protocols. mRNA values were normalized to *IPO8* gene of reference and control (0.1% DMSO) was set to value “1”. Data (Ν = 3) are presented as mean ± SD values. Statistical significance was assessed with Welch’s *t*-test. Asterisks indicate comparisons in between control (0.1% DMSO) and TH301-treated cells, at statistical significance levels of 0.05 and below (*: <0.05; **: <0.01; ***: <0.001). (**B**) γH2AX (p-H2AX-Ser^139^), p21, total p53, and p-p53-Ser^15^ (p53 phosphorylated form at Ser^15^) protein levels in PDAC cells, following treatment with 40 μΜ TH301, for 24 h, examined by Western blotting. (**C**). Relative protein level quantification (γH2AX {p-H2AX-Ser^139^}, p21, p53, and p-p53-Ser^15^), after normalizing densitometry values to *β*-Actin protein of reference, in all 3 PDAC cell lines.

Given that p53 induces *CDKN1A* gene (codes for the p21 protein) transcription [35], we next reasoned to explore whether TH301 impacts the expression of *TP53*/p53 levels in BxPC-3 and PANC-1 cells, considering that the BxPC-3 and PANC-1 cell lines carry point mutations in the *TP53* locus (Table S2), whereas the AsPC-1 cells completely lack both p53 protein expression and *TP53* gene activity (Figure 3). As depicted in Figure 3A, PDAC cell exposure to TH301 for 24 h significantly increased *CDKN1A* levels (>150x; x: induction fold) and reduced mutant *TP53* (*TP53^Y220C^*) contents in BxPC-3 cells, without affecting mutant *TP53* (*TP53^R273H^*) levels in the PANC-1 cellular setting. Of note, *TP53* mRNA contents remained undetectable in AsPC-1 cells (Figure 3A), fully aligning with their lack of p53 protein expression (Figure 3B), either in the presence or in the absence of TH301. Importantly, we also examined p53 protein levels and its phosphorylation status at the critical amino-acid residue of Serine 15 (Ser^15^), alongside the induction of p21 protein, following 24 h of TH301 treatment (40 μM). As shown in Figure 3B,C, the remarkable induction of p21 protein, in response to TH301 administration, proved to operate independently of both total and phosphorylated (p-Ser^15^) p53 protein forms in BxPC-3. PANC-1 cells showed transcriptional-level induction only (Figure 3A). Furthermore, in AsPC-1 cells, p53 (both total and p-Ser^15^) forms are missing, despite the strong transcriptional increase in *CDKN1A* gene activity after TH301 treatment, thereby indicating the p53-independent proficiency of TH301 to strikingly upregulate *CDKN1A*/p21 levels in PDAC environments of diverse mutational loads.

Our findings strongly suggest that TH301 significantly induces *CDKN1A* gene expression, with p21 induction occurring independently of the p53 phosphorylation/activation status, in human pancreatic cancer cells. Next, we evaluated the potential genotoxic effects of TH301 by investigating the phosphorylation levels of Histone H2AX, a well-established marker of DNA damage [36,37,38]. As shown in Figure 3B,C, a notable increase in gamma-H2AX (γH2AX) levels was observed across all three cell lines, following a 24 h exposure to TH301, thereby indicating the capacity of TH301 to exert genotoxic effects in pancreatic cancer cells, in p53-independent manners.

### 2.4. TH301 Induces Caspase Repertoire-Mediated Apoptosis in Pancreatic Cancer Cell Settings: Mutational Signature-Dependent Responses

**Figure 4:**
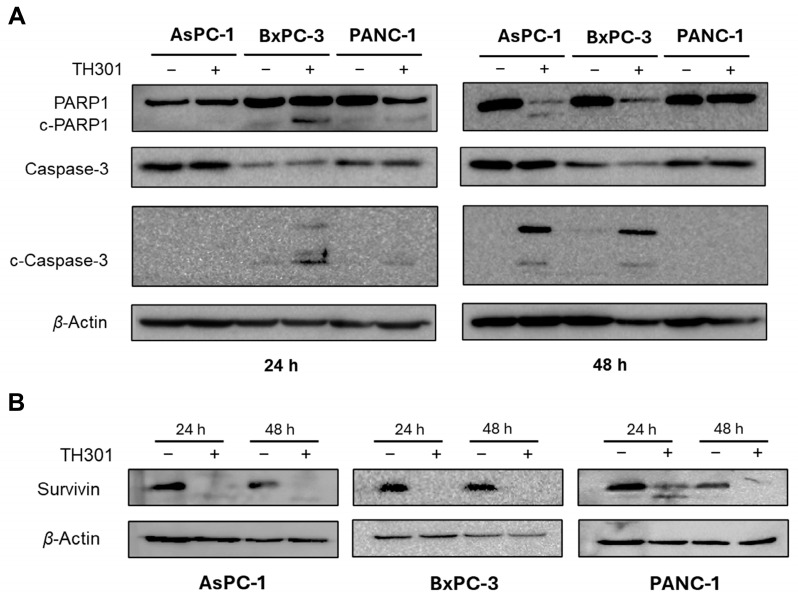
TH301 treatment induces activation of Caspase repertoire-mediated apoptosis and triggers reduction of Survivin expression in human pancreatic cancer cell lines. Western blotting profiles of total and cleaved PARP1 (c-PARP1) and Caspase-3 (c-Caspase-3) (pro-)apoptotic proteins (**A**), and of Survivin anti-apoptotic protein (**B**), in the presence (+) or absence (−) of 40 μΜ TH301, for 24 and 48 h (post-administration), using *β*-Actin as protein of reference (control). “c”: Cleaved.

In addition to cell cycle arrest, the significant decrease in cell viability after exposure of AsPC-1, BxPC-3, and PANC-1 cells to TH301 (Figure 1) could be also associated with activation of apoptosis (Figure 4), a Programmed Cell Death (PCD) major sub-routine. Hence, we examined total protein levels and cleavage patterns of the fundamental apoptosis mediators PARP1 and Caspase-3, after treatment of the three pancreatic cancer cell lines with the TH301 circadian clock modulator, for 24 and 48 h (Figure 4A). Twenty-four hours post-administration, apoptosis activation was readily observed only in BxPC-3 cells, as clearly indicated by the cleaved (“c”) PARP1 (c-PARP1) and Caspase-3 (c-Caspase-3) generated profiles, with the apoptotic program intensified at 48 h of (TH301) treatment. Interestingly, PANC-1 cells were presented as rather tolerant to apoptosis, with only a small sub-population of sensitive cells (c-Caspase-3^+^) undergoing apoptosis after 24 h of TH301 exposure, thus dictating the capacity of TH301 to predominantly exert growth-inhibitory, but not apoptotic, effects on PANC-1 cells (see, Figure 1). In contrast, AsPC-1 cells, in response to their 48 h treatment with TH301, were subjected to Caspase-3-dependent apoptosis (c-Caspase-3^+^/cPARP1^+^), thus providing TH301 with dual properties of both inducing cell cycle arrest and triggering apoptotic death, in specific PDAC mutational settings. Taken together, it seems that TH301 can activate the Caspase-3/PARP1 apoptotic axis in pancreatic cancer cell environments, following a mutational signature-dependent manner. Likewise, TH301 proved able to markedly downregulate the expression levels of Survivin, a key anti-apoptotic protein [39,40,41,42], in all three examined cells lines (Figure 4B), after 24 and 48 h of drug (TH301) treatment, with a novel cleaved fragment easily detected in the 24 h-exposed PANC-1 cells. Its ability to drastically reduce Survivin protein contents in a mutational load-independent fashion enhances TH301 potential to cause PDAC apoptosis and supports its usefulness in novel, promising, and powerful regimens for pancreatic cancer chemotherapy.

### 2.5. TH301 Upregulates LC3B-II-Depenent Autophagy in Pancreatic Cancer Cells, Following a Mutational Signature-Independent Pattern

**Figure 5:**
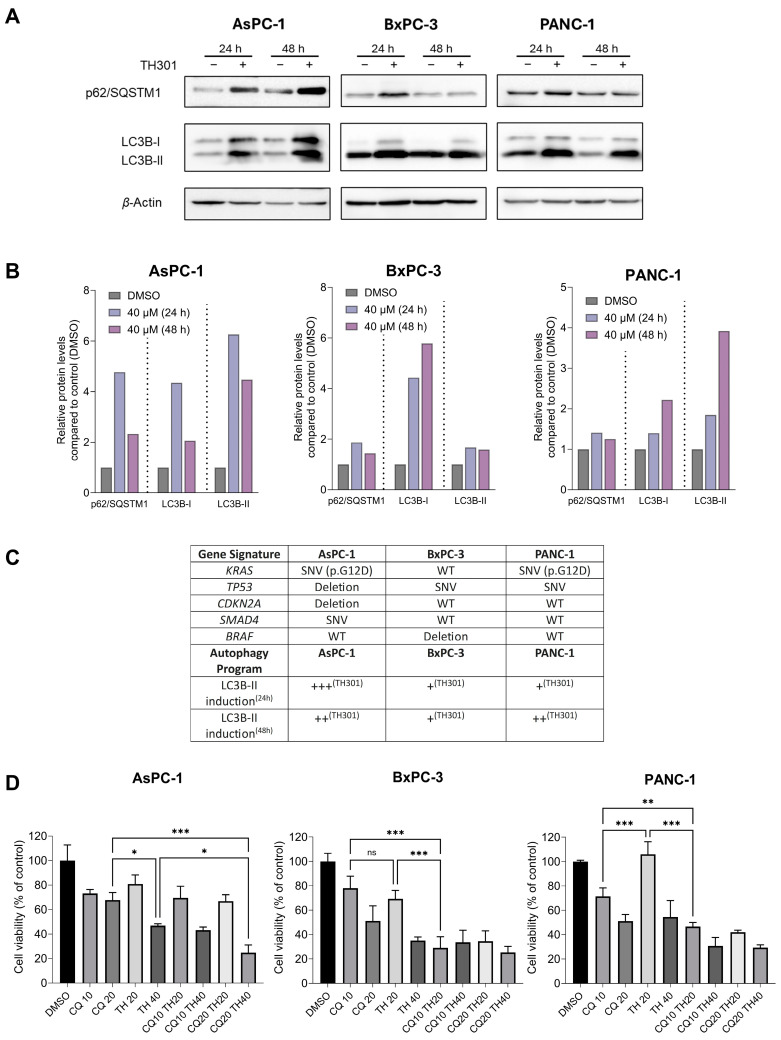
TH301 upregulates autophagy in human pancreatic cancer cells of diverse mutational loads. (**A**) Western blotting profiles of major autophagic markers p62/SQSTM1 and LC3B-II, in presence (+) or absence (−) of 40 μΜ TH301, for 24 and 48 h (post-administration). (**B**) Quantification of “A” conducted via measurements of densitometry values that were normalized to *β*-Actin. Normalized values derived from non-treated cells were set to “1”. Presentation of relative expression of each examined protein, compared to control (0.1% DMSO, showed once), after normalizing densitometry values to *β*-Actin. (**C**) Gene mutation profile and qualitatively characterized LC3B-II induction level in all 3 PDAC cell lines. Major genetic alterations of key mutated genes (*KRAS*, *TP53*, *CDKN2A*, *SMAD4*, and *BRAF*) in the pancreatic ductal adenocarcinoma (PDAC) cell lines AsPC-1, BxPC-3, and PANC-1 are indicated. Mutational data were retrieved from the Dependency Map (DepMap) Portal [48]. LC3B-II induction levels, examined at 24 and 48 h (TH301) post-treatment, are presented as qualitative scores (+++, ++, +) based on LC3B-II protein expression patterns, in the presence or absence of TH301. (**D**) Cell viability profiles (bar-charts) of PDAC cell lines after treatment with CQ (only), TH301 (only), or CQ and TH301 (together), for 48 h (post-administration). Data (N = 3) are presented as mean ± SD values. Statistical significance was assessed with one-way ANOVA and Tukey’s multiple comparison correction (ns: non-significant; *: <0.05; **: <0.01; ***: <0.001).

Autophagy serves as a double-sword process that can play essential roles either in the survival or in the elimination of cancer cells [43,44,45]. To investigate the effects of TH301 on autophagy in pancreatic cancer cell contexts, we treated the AsPC-1, BxPC-3, and PANC-1 cell lines with 40 μΜ TH301, for 24 and 48 h, and analyzed the protein expression levels of the key autophagy markers p62/SQSTM1 and LC3B-II. As described in Figure 5A, in the presence of TH301, there is a significant induction of the LC3B-II protein isoform (and the LC3B-I) in all three pancreatic cancer cell lines examined herein, with a simultaneous elevation of p62/SQSTM1 protein contents also detected, albeit in a time- and cell type-dependent manner (Figure 5A,B), which may indicate either induction of autophagy or impairment of autophagic flux. The mechanistic relationship between mutational profile and induction of LC3B-II by TH301 was also evaluated in the 3 PDAC cell lines. As shown in Figure 5C, TH301 treatment results in a strong, LC3B-II-dependent, upregulation of autophagy, which seems to be critically influenced by the mutational status of key oncogenic mediators, such as the *KRAS* and *BRAF* signaling transducer-coding genes. To further clarify whether the obtained findings reflect an activation of autophagy response, we next co-treated cells with TH301 and Chloroquine (CQ), a known autophagy inhibitor [46,47], and examined cell viability. Of note, the pathogenic effect of CQ alone was evaluated via treatment of cells with CQ increasing concentrations for 48 h, resulting in an IC_50_ range of ~46–18 μM for the three cell lines tested herein (Supplementary Figure S4). Strikingly, our results unveil that TH301 can synergize productively with CQ in BxPC-3 and PANC-1 cells when non-toxic concentrations of both agents are administered together, whereas in AsPC-1 cells, only the 40 μΜ dose of the TH301 clock modulator proves able to synergize with CQ in an effective manner (Figure 5D). Altogether, our results indicate that TH301 can activate an LC3B-II-dependent autophagic program in PDAC cells of diverse mutational loads, and the combination of TH301 with CQ can enhance pancreatic cancer cell pathology and elimination efficiency in a dose-dependent manner. It seems that a novel therapeutic scheme of CQ-sensitizing pancreatic cancer cells to TH301-driven death opens a new window for the simultaneous modulation of autophagy and circadian clock functionalities in the clinical management of the disease.

### 2.6. TH301 Potentiates the Cytopathic Effects of Oxaliplatin by Reducing Pancreatic Cancer Cell Viability

**Figure 6:**
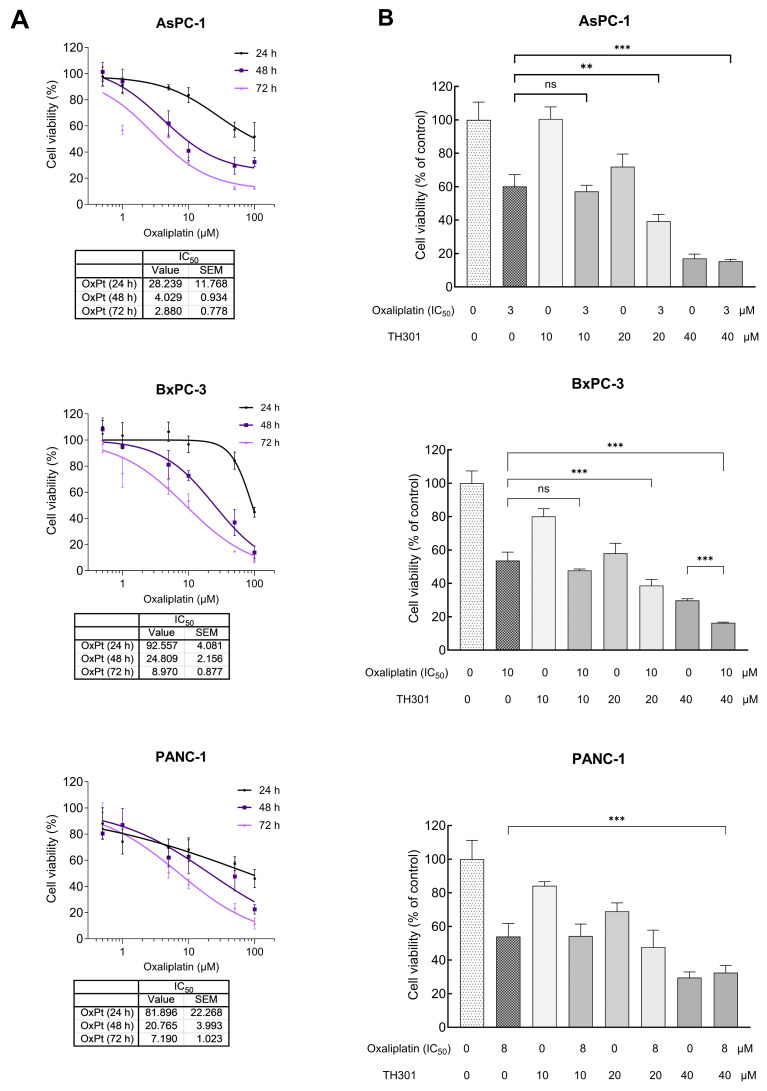
Synergistic cytopathic effects of TH301 and Oxaliplatin agents on human pancreatic cancer cell survival and growth. (**A**) Cell viability curves, after exposure of PDAC (AsPC-1, BxPC-3 and PANC-1) cells to increasing concentrations of Oxaliplatin (OxPt) (0, 1, 10, and 100 μM) for 24, 48, and 72 h, with the obtained IC_50_ values indicated, respectively. Data (N = 4) are presented as mean ± SEM values. IC_50_ values were calculated with a non-linear model platform. (**B**) Cell viability quantification was performed through MTT assay engagement, after treatment of PDAC (AsPC-1, BxPC-3, and PANC-1) cells with increasing concentrations of TH301 (0, 10, 20, and 40 μΜ), in the presence (+) or absence (−) of OxPt (IC_50_ concentration) for 72 h. Data (N = 4) are presented as mean ± SEM values. Statistical significance was assessed with one-way ANOVA and Tukey’s multiple comparison correction. Asterisks indicate statistical significance (ns: non-significant; **: <0.01; ***: < 0.001).

Oxaliplatin is utilized in the treatment of PDAC, as critical component of combination chemotherapy regimens, such as the FOLFIRINOX (Folinic Acid, Fluorouracil, Irinotecan and Oxaliplatin) drug cocktail. Given that Oxaliplatin inhibits cell proliferation, growth, and survival, by inducing DNA damage, we aimed herein to investigate whether TH301 could synergize with Oxaliplatin to further reduce cell viability of PDAC cells. After identifying the IC_50_ value, for each one of the three cell lines (Figure 6A), we combined this Oxaliplatin concentration with 10, 20, and 40 μM of TH301, respectively, to treat PDAC cells for 72 h (post-administration), and cell viability was next quantified though MTT assay engagement. As illustrated in Figure 6B, co-treatment with TH301 and Oxaliplatin led to their significant synergism, in reducing cell viability, particularly for the AsPC-1 and BxPC-3 cell lines. However, the synergistic effect was missing from PANC-1 cells, likely due to their specific mutational content that can render them comparatively “semi-tolerant” to the TH301—Oxaliplatin cocktail scheme applied herein. Altogether, a novel regimen containing non-toxic doses of the TH301 and Oxaliplatin agents seems to hold strong therapeutic promise for pancreatic cancer in the clinical setting.

### 2.7. CRY2 and BMAL1 Are Not Required for the TH301-Driven Induction of p21 Cell Cycle Inhibitor in Pancreatic Cancer Cells

**Figure 7:**
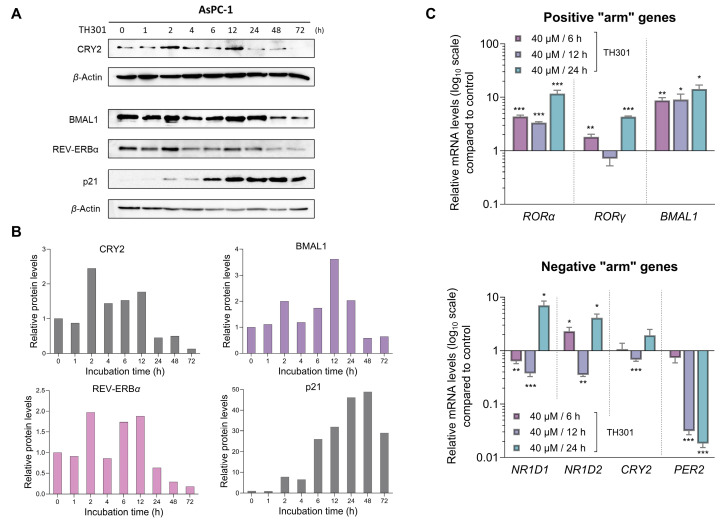
TH301 modulates core clock proteins, while it induces p21 expression in PDAC cell settings. (**A**) Western blotting profiles of the major circadian clock proteins CRY2, BMAL1, and REV-ERBα, and the cardinal cell cycle regulator p21, after treatment of AsPC-1 cells with 40 μΜ TH301 for 0–72 h. (**B**) Quantification of relative protein levels, after normalizing densitometry values to *β*-Actin. Normalized treated cell values for the time point t = 0 h were set to value “1”. (**C**) mRNA levels of the clock genes *RORα*, *RORγ*, *BMAL1* (top bar-chart), *NR1D1*, *NR1D2*, *CRY2*, and *PER2* (bottom bar-chart) in AsPC-1 cells, following treatment with 40 μΜ TH301 for 6, 12, and 24 h, detected and quantified by RT-qPCR protocols. mRNA values were normalized to *IPO8* respective one, while control (0.1% DMSO) was set to value “1”. Data (Ν = 3) are presented as mean ± SD values. Statistical significance was assessed with Welch’s *t*-test, as each time point was considered an independent experiment. Asterisks indicate comparisons in between control (0.1% DMSO) and TH301-treated cells, at significance levels of 0.05 and below (*: <0.05; **: <0.01; ***: <0.001).

Since TH301 is known to stabilize CRY2, we next sought to investigate the effects of TH301 on the expression of circadian clock key components, which are integral determinants to both the “positive” and the “negative” arm of the circadian clock feedback loop (Figure 7). Hence, we utilized AsPC-1 cells, since they presented the most pronounced cytopathic responses to TH301 treatment, as compared to the other two cell lines analyzed herein (see Figure 1 and Figure 2). Most importantly, AsPC-1 cells completely lack *TP53*/p53 expression and activity (see Figure 3), allowing us to minimize confounding factors, such as p53, which have been previously reported to influence, and be influenced by, the circadian clock components [49,50]. We first examined the expression levels of *RORα*, *RORγ*, *BMAL1* (positive “arm”), *NR1D1*, *NR1D2*, *CRY2*, and *PER2* (negative “arm”) genes, with *RORα* and *BMAL1* of the positive “arm” presented with a notable upregulation in response to TH301 administration (Figure 7C; top bar-chart). On the contrary, the *PER2* gene that belongs to the negative “arm” of the clock exhibited drastic reduction in its transcriptional activity, following a time-dependent pattern, in the presence of TH301 (Figure 7C; bottom bar-chart). Likewise, *PER2* was subjected to a strong transcriptional downregulation, after TH301 administration, in BxPC-3 and PANC-1 cells (Supplementary Figure S5), thus indicating the mutational load-independent association of *PER2* and TH301 in PDAC settings. Furthermore, to explore the potential correlation of TH301-mediated modulation of the circadian clock with the induction of p21 cell cycle inhibitor, we analyzed and quantified the expression levels of CRY2, BMAL1, REV-ERBα, and p21 proteins, after treating AsPC-1 cells with 40 μM TH301, for various time periods (0–72 h). Figure 7A,B describe the ability of TH301 to cause detectable elevations of CRY2, BMAL1, and REV-ERBα protein contents, albeit at different time points of the treatment period. Although the levels of circadian clock proteins are significantly decreased after 48 and 72 h of treatment, the p21 protein expression was presented with a striking increase, in response to TH301, at the same time points and even earlier (12–72 h), thus dictating the mechanistic uncoupling of CRY2 and BMAL1 activities from p21 protein induction. Altogether, our findings prove the proficiency of TH301 (a) to perturb circadian clock integrity (e.g., via *PER2* transcriptional suppression) and (b) to induce cell cycle arrest, via a striking increase in p21 protein (and gene) levels, in human pancreatic cancer cells of specific mutational signatures.

**Figure 8:**
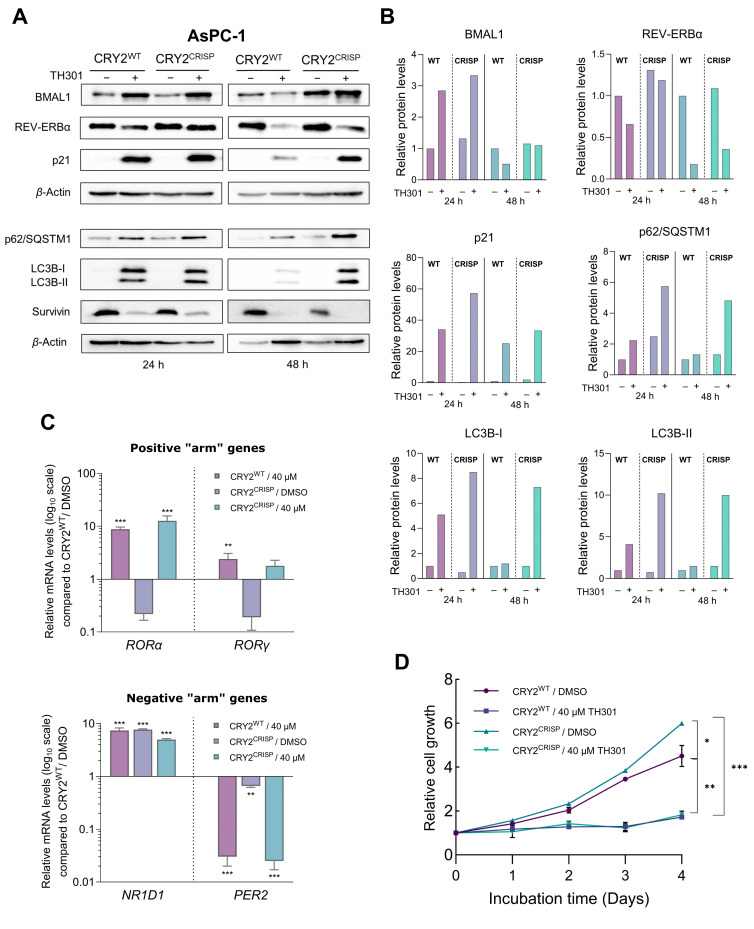
CRISPR/Cas9-mediated targeting of CRY2 does not affect p21 induction by TH301 in pancreatic cancer cells. (**A**) Western blotting of (a) clock proteins BMAL1 and REV-ERBα, (b) autophagy markers p62/SQSTM1 and LC3B-II (and LC3B-I), (c) cell cycle regulator p21, and (d) apoptosis inhibitor Survivin, after treating wild-type (CRY2^WT^) and targeted heterozygous (CRY2^CRISP^) AsPC-1 cells with 40 μΜ TH301 for 24 and 48 h. (**B**) Relative protein levels after normalization of densitometry values to *β*-Actin. Normalized CRY2^WT^ values, for 24 and 48 h, were set to value “1”. (**C**) Quantification of mRNA transcript levels of the clock genes *RORα* and *RORγ* (positive “arm”), and *NR1D1* and *PER2* (negative “arm”), in CRY2^WT^ and CRY2^CRISP^ AsPC-1 cells, following treatment with 40 μΜ TH301 for 24 h, conducted by RT-qPCR protocol. *GAPDH* served as gene of reference, and CRY2^WT^ control (0.1% DMSO) was set to value “1”. Data (Ν = 3) are presented as mean ± SD values. Statistical significance was assessed via one-way ANOVA, with Dunnet’s multiple comparison correction. Asterisks indicate comparisons of control (CRY2^WT^, 0.1% DMSO; not shown) to treated CRY2^WT^, non-treated and treated heterozygous CRY2^CRISP^ AsPC-1 cells, at significance levels of “0.05” and below values (**: <0.01; ***: <0.001). (**D**) Relative cell growth rates of CRY2^WT^ and CRY2^CRISP^ AsPC-1 cells, in the absence or presence of 40 μΜ TH301 for 0–4 days. Data (N = 4) are presented as mean ± SD values. Statistical significance was assessed with one-way ANOVA, via Tukey’s multiple comparison correction. Asterisks indicate comparisons in between paired groups, after 0–4 days of TH301 exposure (*: <0.05; **: <0.01; ***: <0.001).

Given that TH301 can cause time-dependent alterations in CRY2 and BMAL1 expression, together with concurrent p21 induction, we next sought to further investigate the potential relationship between the modulation in protein levels of CRY2 or BMAL1 clock components and the increase of p21 contents in the presence of TH301. To explore this, we attempted herein to knock-out *CRY2* or knock-down *BMAL1* in AsPC-1 cells. Following these gene-targeting protocols, we treated cells with TH301 and analyzed p21 protein levels for all experimental conditions applied in AsPC-1 cells (i.e., CRY2^WT^ and CRY2^CRISP^, in the absence and presence of TH301). For the partial loss of *CRY2*, we employed a dual gRNA-CRISPR/Cas9 Lentiviral system, which resulted in a ~3.4 Kb deletion, encompassing Exons 3–5 of the *CRY2* gene locus (Supplementary Figure S6). Of note, we were unable to isolate homozygous clones for the deletion, likely due to the essential role(s) of CRY protein in pancreatic cancer cell survival and growth, and thus we proceeded with the heterozygous AsPC-1 cells for further analysis. As described in Figure 8A, partial loss of CRY2 (CRY2^CRISP^) could not affect the TH301-driven induction of p21 protein, as compared to control (CRY2^WT^) cells. Likewise, CRY2^CRISP^ (AsPC-1) cells were not presented with major perturbations in the expression profiles of BMAL1 and REV-ERBα proteins, and of the circadian clock genes *RORα*, *RORγ*, *NR1D1*, and *PER2* (Figure 8A,C). Our findings strongly suggest that the TH301-induced modulation of the circadian clock components and the induction of p21 protein are two independent phenomena.

Next, we questioned whether the partial loss of CRY2 could affect the TH301-induced activation of autophagy components and reduction of Survivin contents. It seems that, in contrast to Survivin’s profile (decreased levels, in response to TH301) that remained unaffected, for both control (CRY2^WT^) and targeted (CRY2^CRISP^) cells, the two key markers of autophagy, p62/SQSTM1 and LC3B-II (and LC3B-I), were shown with similarly increased protein contents both in CRY2^CRISP^ and in CRY2^WT^ (AsPC-1) cells after their exposure (24 and 48 h) to the TH301 agent (Figure 8A,Β). Interestingly, treatment of CRY2^WT^ and CRY2^CRISP^ (AsPC-1) cells with TH301 for 4 consecutive days resulted in significantly reduced cell growth rates for both cell types (Figure 8D), although TH301-untreated CRY2^CRISP^ cells showed a slightly increased growth rate compared to their CRY2^WT^ counterparts.

Taken together, our results strongly suggest that TH301 exerts opposite actions on p21 (remarkable induction) and Survivin (notable reduction) protein expression patterns that occur independently of CRY2 modulation in human pancreatic cancer cell settings. Nevertheless, a “gene dose-specific effect”, with the *CRY2*-gene knock-out homozygosity, but not heterozygosity, likely indispensable for presumable development of severe impairments in gene transcription activities, upon PDAC cell exposure to TH301, cannot be excluded, and thus requires further exploration.

**Figure 9:**
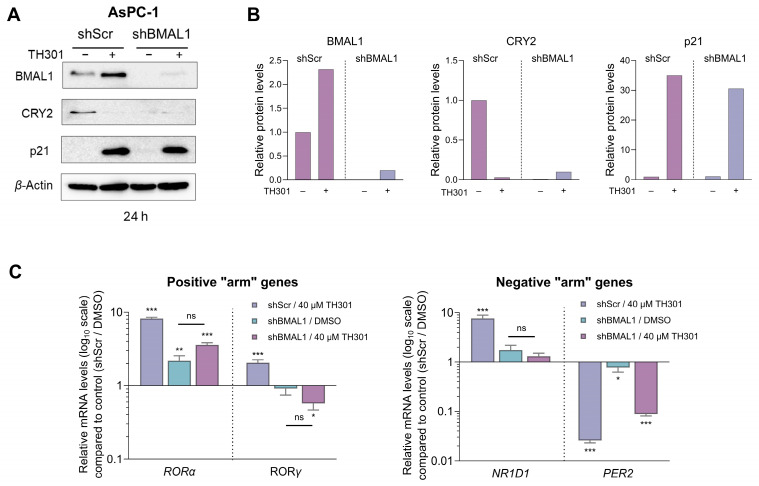
shRNA-mediated BMAL1 knock-down does not affect p21 induction by TH301 in pancreatic cancer cells. (**A**) Western blotting of (a) clock proteins BMAL1 and CRY2 and (b) cell cycle regulator p21, after treating control (shScr) and BMAL1 knock-down (shBMAL1) AsPC-1 cells, with 40 μΜ TH301 for 24 h. (**B**) Relative protein levels, after normalizing densitometry values to *β*-Actin. Normalized shScr values were set to value “1”. (**C**) Quantification of mRNA transcript levels of the clock genes *RORα*, *RORγ*, *NR1D1*, and *PER2*, in shScr and shBMAL1 AsPC-1 cells, following treatment with 40 μΜ TH301 for 24 h, performed by RT-qPCR protocols. mRNA values were normalized to *GAPDH*, while control (shScr, 0.1% DMSO) was set to value “1”. Data (Ν = 3) are presented as mean ± SD values. Statistical significance was assessed via one-way ANOVA, with Dunnet’s multiple comparison correction. Asterisks indicate paired comparisons between control shScr (0.1% DMSO), (TH301) treated shScr, non-treated and TH301-treated shBMAL1 AsPC-1 cells, at significance levels of “0.05” and below values (ns: non-significant; *: <0.05; **: <0.01; ***: <0.001).

Since we observed a notable TH301-induced increase of its protein contents 24 h post-administration, BMAL1 was next examined for a presumable functional association with the p21 cell cycle inhibitor (Figure 9). Hence, we specifically knocked-down BMAL1 in AsPC-1 cells, through employment of the shRNA-based technology (Supplementary Figure S7), and subsequently determined p21 protein levels. As shown in Figure 9A,B, knock-down of BMAL1 (shBMAL1) proved unable to affect the TH301-mediated induction of p21 protein contents. Interestingly, CRY2 levels were significantly reduced in untreated BMAL1 knock-down (shBMAL1) cells, and did not increase after TH301 treatment, thereby suggesting the lack of any mechanistic correlation between BMAL1 or CRY2 with p21 induction in response to TH301 clock modulator. Of note, transcriptional activities of other critical clock genes examined herein, such as the *RORα*, *RORγ*, *NR1D1*, and *PER2* ones, in BMAL1 knock-down (shBMAL1) cells, were not significantly affected by TH301 administration, compared to Scramble (control) (shScr) cells. Taken together, our findings reveal, for the first time, the BMAL1-independent induction of p21 protein in response to TH301, and demonstrate that modulation of the circadian clock components cannot influence p21 upregulation by TH301 in human pancreatic cancer cell environments.

## 3. Discussion

Modulating cell viability and cell cycle progression is a valuable target for treating malignancies, as sustained proliferation and cell cycle regulation abnormalities are hallmarks of cancer [51,52]. In this work, we have evaluated the circadian clock modulator TH301 for its anti-cancer properties in the three human PDAC cell lines AsPC-1, BxPC-3, and PANC-1. Our results show that TH301 exerts anti-proliferative effects, selectively induces apoptosis, and synergizes with Chloroquine or Oxaliplatin to further reduce PDAC cell viability. Notably, the remarkable increase of p21 expression is presented herein as a CRY2- and BMAL1-independent response that is induced by TH301, thereby suggesting that TH301 can affect essential cellular pathways beyond the circadian clock. Interestingly, our three PDAC cell lines stemmed from distinct clinical settings that could influence their biological behavior and response to treatment. AsPC-1 cells are derived from metastatic tissue following chemotherapy and radiation, and thus represent a treatment-experienced and highly aggressive phenotype. In contrast, BxPC-3 cells originate from a localized tumor without evidence of metastasis, whereas PANC-1 cells are generated from a primary tumor with limited metastatic progression to peri-pancreatic lymph nodes [53,54,55]. Despite these differences in origin and presumed clinical aggressiveness, our results demonstrate that TH301 can exert its anti-oncogenic power in a manner that acts independently of PDAC cells’ tissue origin or prior treatment history. However, a drug-facilitated sensitization process ensued by a TH301-based treatment may prove to be a novel and beneficial regimen for the metastatic form of the disease, as it can be likely foreseen by the differential cell viability profiles of AsPC-1 as compared to the BxPC-3 and PANC-1 cells, in response to the TH301 agent (Figure 1B).

Our MTT cytotoxicity assays performed with the TH301 pharmacological concentration of IC_50_ at 40 μM revealed a significant decrease of human pancreatic cancer cell survival, in a dose- and time-dependent manner, with AsPC-1 and BxPC-3 cells exhibiting higher sensitivities compared to the PANC-1 cells. Importantly, the mechanism underlying this cell viability reduction seems to be linked to the induction of a G1-phase cell cycle arrest, as documented by the significant accumulation of cells at the G1-phase after TH301 treatment, coupled with a decrease in the S-phase population, thus indicating the inhibition of DNA synthesis and subsequent cell cycle progression. In accordance, the marked decrease of Cyclin D3 protein levels and increase of p27 protein contents that are detected in all three cell lines strongly suggest a common mechanism of TH301 action in pancreatic cancer cells of diverse mutational composition. Likewise, p21 also serves as a major inducible biomarker of TH301 anti-cancer activity, specifically in the AsPC-1 and BxPC-3 PDAC cell lines. Previous research has highlighted the critical role of p21 in mediating G1-arrest by inhibiting the cell cycle-promoting activity of CDK–Cyclin complexes, thus preventing the phosphorylation of Retinoblastoma protein (Rb) and blocking the subsequent cell cycle progression [56,57]. Intriguingly, PANC-1 cells presented with a lack of p21 induction, despite the increase of its *CDKN1A* mRNA, in response to TH301, which could be associated with post-translational modifications directly compromising protein’s stability [58]. Hence, in the PANC-1 cellular setting, G1-arrest may occur via a p27 upregulation, but in a p21-independent fashion, as previously reported for other systems [59]. Cell cycle typically relies on Cyclins and Cyclin-Dependent Kinases (CDKs), as well as on their functional control by CDK Inhibitors (CDKIs: KIP/CIP and INK4 protein families), to drive the progression through its phases; molecular processes that are usually disrupted in cancer [51,52]. Targeting these deregulated cell cycle proteins has emerged as a promising strategy for inhibiting tumor growth. Development of clinically successful CDK4/6 inhibitors (CDK4/6i) like Palbociclib (PD 0332991), which can cause G1-phase arrest by targeting the Cyclin D-CDK4/6 complexes, has been a significant breakthrough in cancer treatment [60]. Similarly, our results demonstrate a comparable anti-proliferative effect of TH301 on all three PDAC cell lines examined by inducing G1-phase cell cycle arrest, and modulating the expression of key cell cycle regulators, including Cyclin D3, CDK4, CDK6, CDK2, p21, and p27, in dose- and cell type-dependent manners. Of note, the significant reduction in the expression of stemness genes, particularly *NANOG*, across all three cell lines, along with the *SOX2* and *OCT4* downregulation in the BxPC-3 and PANC-1 cell types, unveils the capacity of TH301 to not only inhibit proliferation but to also reduce stemness (or stem-like properties) of PDAC cells, with the stemness phenotype frequently associated with resistance to conventional therapies [61]. These molecular changes underscore the striking ability of TH301 to interfere with both cell cycle and stemness machineries, thereby inhibiting cancer cell proliferation and overcoming cancer stemness-mediated resistance.

The striking induction of *CDKN1A*/p21 by TH301, independently of the *TP53*/p53 status, in diverse PDAC cellular environments represents another novel and important finding of our study. *TP53*-gene mutations in PDAC are often associated with “Gain-Of-Function” (GOF) activities that promote cancer cell proliferation and survival [62,63,64,65,66]. *CDKN1A*/p21 can be induced independently of p53 via activation of distinct transcription factors, such as the STAT (Signal Transducer and Activator of Transcription) family members, Sp1, and others, in response to different stimuli [67,68,69]. Our results prove that the TH301-induced p21 upregulation can bypass the canonical p53 pathway, thereby providing an effective strategy to counteract the proliferation of PDAC cells harboring GOF p53-mutant forms. The observed reduction in mutant *TP53*/p53 levels in BxPC-3 and PANC-1 cells by TH301 may indicate the role of TH301 in the transcriptional repression of *TP53* gene, and/or in the destabilization of mutant p53 protein. This highlights a potentially dual action of TH301: inducing *CDKN1A*/p21 independently of p53, while simultaneously suppressing mutant *TP53*/p53 levels. Future studies should investigate the mechanistic basis of TH301-induced *TP53*/p53 reduction, including the effects of TH301 on mutant p53 proteasomal degradation and/or mutant *TP53* transcriptional suppression. Interestingly, the ability of TH301 to increase the γH2AX protein levels strongly suggests that TH301 treatment can promote genotoxic stress in a p53-independent manner. Whether there is novel crosstalk in between GOF p53-mutant activity and PDAC genotoxicity in the presence of TH301 remains elusive, and needs to be further investigated in the future.

Induction of apoptosis, as evidenced by the proteolytic cleavage of PARP1 (inactivation) and Caspase-3 (activation) in the BxPC-3 cells, and, to a lesser extent, in the AsPC-1 (magnified at 48 h) and PANC-1 (hardly detectable) cell lines, along with the severe reduction in Survivin protein contents in response to TH301 exposure in all three (PDAC) cell lines examined herein, supports the (pro-)apoptotic capacity of TH301 in human pancreatic cancer cells of diverse mutational background. Survivin, a member of the Inhibitor of APoptosis (IAP) family, is known to play a crucial role in inhibiting apoptosis and promoting proliferation of cancer cells, including PDAC [41,42]. Downregulation of Survivin by TH301 indicates the compound’s power to effectively counteract major survival mechanisms employed by human PDAC cells of diverse mutational signatures.

Another key survival mechanism in PDAC is the elevated autophagic activity, which serves, among others, to control ROS (Reactive Oxygen Species) production and, ultimately, DNA damage, thus allowing for continued tumor growth [45]. The significant increase in LC3B-II (and LC3B-I) protein levels and the notable accumulation of p62/SQSTM1 protein after TH301 administration unveil the proficiency of TH301 to enhance basal autophagy in PDAC cells of different oncogenic potential. The TH301 (super)-induced autophagy may rely on mutant KRAS^G12D^ oncogenic signaling for effective activation of the autophagic machinery. Furthermore, it seems that wild-type BRAF kinase is essential to maintain signaling integrity and enable robust LC3B-II upregulation. In contrast, BxPC-3 cells, which lack BRAF, display a markedly reduced autophagic response, highlighting that BRAF loss likely compromise autophagy (super-)activation by exposure to TH301. Remarkably, p53 is not required for LC3B-II-mediated autophagy induction, as evidenced by the strong LC3B-II levels in p53-deficient AsPC-1 cells after TH301 administration. These results underline the critical role of BRAF/KRAS signaling axis in driving LC3B-II-dependent autophagy in TH301-treated PDAC cells. This aligns with prior reports linking RAS activation to autophagy regulation [70]. Combination of TH301 with Chloroquine (CQ), an autophagy inhibitor, further augmented cytotoxicity, thus suggesting that TH301-induced autophagy likely serves as a protective mechanism of pancreatic cancer cells undergoing TH301-driven apoptotic death. Chloroquine treatment alone (single-drug scheme), or in combination with chemotherapy agents (cocktail-drug schemes), have already been shown to attenuate PDAC cell growth [47]. In our study, Chloroquine sensitized PDAC cells to TH301, thereby resulting in increased cell death. This synergistic effect highlights the potential of combining TH301 with autophagy inhibitors as a novel and promising therapeutic strategy to overcome PDAC resistance and improve treatment efficacy. Our findings suggest that TH301 could be successfully evaluated in vivo, as a new and targeted anti-cancer agent, for efficiently disrupting PDAC proliferation and substantially strengthening existing therapeutic regimens in clinical settings.

Previous studies have reported the anti-tumorigenic and chemotherapeutic effects of circadian clock-modulating agents alone or in combination with known anti-cancer drugs. In this context, the majority of analyzed compounds were agonists of REV-ERBα, REV-ERBβ, and RORα proteins [71,72,73,74,75]. Regarding the CRY family members, most of the existing synthetic stabilizers can act either specifically for CRY1 [33,76] or are unable to selectively target either isoform, which remains a major drawback for new generation treatments as each CRY isoform has been shown to carry distinct functions in the clock and beyond [29,30,31,32,77,78]. Concerning CRY2, to our knowledge, there are no reports to date describing any anti-cancer activity of the only existing CRY2-specific stabilizer, TH301. Recently, Miller and colleagues demonstrated that TH301 binds to the FAD-binding pocket of CRY2, thereby inhibiting FBXL3-mediated degradation and selectively stabilizing the CRY2 isoform. Using BMAL1:dluc and PER2:dluc reporters in synchronized cells, they also showed that TH301 dampens circadian clock’s oscillatory strength/amplitude, particularly after 24 h of continuous TH301 treatment [33]. Although our experiments involved non-synchronized cell cultures and comparatively higher concentrations of TH301, our findings are consistent with their results, as we observed marked modulation of core clock proteins that is typified by a notable decrease in their expression after longer than 24 h-exposure time to the TH301 agent (i.e., 24–72 h).

Surprisingly, our discoveries extend these data by demonstrating that TH301-derived effects on p53-independent p21 induction, LC3B-II-dependent autophagy upregulation, and Survivin protein reduction constitute complex processes, and likely involve additional critical pathways beyond CRY2 modulation. BMAL1 has also been suggested to play a tumor-suppressor role in PDAC, as its overexpression can cause cell cycle arrest and apoptosis, most probably through a p53-dependent pathway [79]. In contrast, our results show that TH301-mediated BMAL1 upregulation, or its genetic silencing, cannot affect the TH301-induced increase in p21 protein levels. The uncoupling of p21 induction from *BMAL1*- and *CRY2*-gene functions strongly suggests that TH301 can activate and upregulate p21 cell cycle inhibitor through a different, yet unidentified, mechanism that is not directly linked to the core clock machinery. This independence could be attributed to the lack of wild-type p53, since it has been shown to serve as one of the critical components that couples the circadian clock to the cell cycle oscillator [80]. Interestingly, Ozturk and colleagues demonstrated that loss of CRYs offers a protective effect against cancer in mutant p53 mice, following genotoxic stress, thus indicating a presumable connection and crosstalk in between CRYs and p53 functionalities [81]. We unveil that TH301 causes severe reduction in CRY2 expression levels, most likely sensitizing the mutant p53 PDAC cell types examined herein to the administered compound TH301. Nevertheless, the partial loss of CRY2 alone could only affect the circadian clock-machinery activities, without significantly altering the phenotypic responses of AsPC-1 cells to the TH301 agent. Taken together, it seems that pharmacological targeting with TH301 deregulates pathways that function independently and beyond the circadian clock, and are critically implicated in cell cycle arrest and apoptosis activation.

The examination and evaluation of the circadian clock modulator TH301 as an anti-cancer agent, which are thoroughly described in the present work, have uncovered several promising avenues for the treatment of Pancreatic Ductal Adeno-Carcinoma (PDAC) that, despite strenuous efforts, still remains one of the most lethal malignancies [1]. The timeline of *CDKN1A* gene activation observed in PDAC-treated cells indicates the involvement of a novel regulatory mechanism orchestrating (*CDKN1A*) gene transcription, in response to TH301. This suggests a new role for TH301 as a chemical regulator of transcription factors directly influencing *CDKN1A* gene activity, beyond the known function of TH301 as a circadian clock modulator [33]. A proposed molecular model, functionally integrating our findings, mainly focuses on the partial structural resemblance of TH301 with several MYC protein inhibitors [82,83]. Providing their similarities in 2D (two-dimensional) and 3D (three-dimensional) structures, as well as mode of actions, TH301 could interfere with MYC activity, thereby alleviating its repressive effect on *CDKN1A* gene transcription [69]. This presumable inhibition of MYC activity could explain the strong and rapid activation of *CDKN1A* gene detected herein in response to TH301 administration. Currently, we are investigating the capacity of TH301 to inhibit MYC function(s) and subsequently derepress *CDKN1A* gene transcription. Taken together, our efforts aim to further elucidate the mechanisms underlying TH301’s transcriptional regulatory effects, highlighting its potential as a versatile and multi-faceted anti-cancer agent, with roles extending beyond circadian clock targeting.

Of note, the capacity of TH301 to compromise stemness and to induce apoptosis, particularly in AsPC-1 and BxPC-3 cells, further enhances its chemotherapeutic power in PDAC environments. Moreover, its ability to severely diminish Survivin protein expression, and to strongly induce autophagy program, indicates that TH301 can critically impact multiple survival pathways in PDAC cellular settings, thereby rendering TH301 a multifaceted and versatile agent, with novel anti-proliferative, anti-survival, and anti-oncogenic properties against human PDACs of diverse mutational signatures.

## 4. Materials and Methods

### 4.1. Cell Lines and Culture Conditions

The human pancreatic cancer cell lines BxPC-3 and PANC-1 were kindly and generously obtained from Professor Karamouzis (Faculty of Medicine, National and Kapodistrian University of Athens, Athens, Greece), while the human pancreatic cancer cell line AsPC-1 was kindly provided by Professor Dimas (Faculty of Medicine, University of Thessaly, Greece). HEK-293T cells were purchased from the American Type Culture Collection (ATCC, Manassas, VA, USA). The AsPC-1 cell line was derived from a 62-year-old woman with pancreatic head adenocarcinoma and widespread abdominal metastases. The patient, treated with radiation and chemotherapy, developed ascites, from which the cell line was established, producing abundant mucin and carcinoembryonic antigen [53]. The BxPC-3 cell line stemmed from a 61-year-old woman’s pancreatic body adenocarcinoma, with no evidence of metastasis. Despite radiation and chemotherapy, the patient died six months later. Tumors in nude mice resembled the primary tumor, producing carcinoembryonic antigen, pancreas-associated antigens, and traces of mucin [54]. The PANC-1 cell line originated from a 56-year-old male with pancreatic head adenocarcinoma, invading the duodenal wall and metastasizing to one peri-pancreatic lymph node. The cell line does not secrete significant carcinoembryonic antigen in culture [55]. All cell lines were cultured according to each provider’s recommendations. More specifically, AsPC-1 and BxPC-3 cell lines were cultured in Roswell Park Memorial Institute 1640 medium (1× RPMI-1640, ATCC modification; Gibco—Thermo Fisher Scientific, Waltham, MA, USA). PANC-1 and HEK-293T cell lines were cultured in Dulbecco’s Modified Eagle Medium (1× DMEM, supplemented with High Glucose, Stable Glutamine and Sodium Pyruvate; Biosera, Cholet, France). Both media were supplemented with 10% Fetal Bovine Serum (FBS; Gibco—Thermo Fisher Scientific), 2.5% Sodium Bicarbonate (Biowest, Nuaillé, France), 1% Non-essential Amino-acids (Biowest), 100 U/mL Penicillin-Streptomycin (Biowest), and 2.5 μg/mL Amphotericin B (Biowest). All cells were maintained at +37 °C in a humidified air-atmosphere (>95% humidity) and 5% CO_2_ environment.

### 4.2. Inhibitors

KS15 (Aobious Inc., Gloucester, MA, USA), KL001 (Cayman Chemical Company, Ann Arbor, MI, USA), TH301 (Focus Biomolecules, Plymouth Meeting, PA, USA), and Chloroquine (TargetMol Chemicals Inc., Boston, MA, USA) chemical compounds (inhibitors) were dissolved in 100% dimethyl sulfoxide (DMSO; Sigma-Aldrich, St. Louis, MO, USA) at a stock concentration of 0.1 M for KL001 and TH301, 0.05 M for KS15, and 10 mM for Chloroquine. Stock solutions were stored at −20 °C in single-use aliquots. For cell treatment, each reagent was diluted in the appropriate culture medium to a final DMSO concentration of 0.1%. Oxaliplatin (Intas Pharmaceuticals Ltd., Ahmedabad, Gujarat, India) was diluted in water for injection at a final concentration of 0.01 M and used immediately. All solutions were thoroughly mixed before being added to the cells.

### 4.3. MTT Assay

Inhibitor-induced cytotoxicity and cell growth arrest were quantified by the MTT assay. Briefly, cells were plated in 96-well flat-bottomed micro-plates, containing 100 μL culture medium, at a density of approximately 5000 cells/well for cytotoxicity and 1500 cells/well for cell growth arrest measurements. Seeded cells were incubated overnight at +37 °C in a humidified atmosphere (>95%) and 5% CO_2_ environment. After incubation, the old medium was replaced with fresh medium, containing appropriate concentrations of reagents/inhibitors. At specific time points of treatment, medium from each well was replaced with Thiazolyl Blue Tetrazolium Bromide (MTT; Abcam, Cambridge, UK), dissolved at a final concentration of 1 mg/mL, in serum-free and phenol-red-free DMEM medium (PAN Biotech, Aidenbach, Germany), and maintained for another 4 h. Then, the MTT-Formazan product was solubilized in Isopropanol, and the optical density was measured with a Spark Multimode Micro-plate Reader (TECAN Group Ltd., Männedorf, Switzerland) at a wavelength of 550 nm and a reference wavelength of 690 nm.

### 4.4. Cell Cycle Analysis

A total of 1 × 10^6^ cells were seeded in 6 cm cell culture dishes and incubated over-night, at +37 °C and 5% CO_2_, in a humidified atmosphere (>95% humidity). Following treatment, cells were mildly collected with cell scraper, centrifuged at 300× *g* for 5 min, and washed in Phosphate-Buffered Saline (PBS; 1×). Next, cells were fixed in 70% ice-cold Ethanol for at least 30 min at +4 °C, washed with 1× PBS, and re-suspended in DNA staining solution, containing 50 mg/mL Propidium Iodide (PI; Sigma-Aldrich, St. Louis, MO, USA), 10 μg/mL RNAse A (in 1× PBS), 5 mM MgCl_2_ (1 M Stock), and 10 mM Tris-HCl (pH 7.5; 1 M Stock), and incubated for 30 min in the dark at room temperature (RT). Cells were analyzed by the Flow Cytometer Cytomics FC 500 (Beckman-Coulter, Brea, CA, USA), using the advanced CXP software v2.2 (Beckman-Coulter).

### 4.5. RNA Extraction

Cells were plated in 6 cm culture dishes, and incubated at +37 °C and 5% CO_2_ (>95% humidity), until they reached ~90% confluency. At specific time points following inhibitor administration, and/or transfection/transduction, cells were collected by mild scraping, then centrifuged at 1500× *g* for 5 min, with the obtained cell pellets washed twice in 1× PBS. Total RNA was isolated with the FastGene RNA Premium Kit (Nippon Genetics, Tokyo, Japan), following manufacturer’s instructions. Final RNA concentration was measured using a high-accuracy Spectrophotometer NanoDrop ND-1000 UV-Vis (Thermo Scientific, Waltham, MA, USA) and RNA was stored at −80 °C for further use.

### 4.6. RT-qPCR

For RT-qPCR (real-time quantitative polymerase chain reaction) analysis, the extracted RNA was reverse transcribed into cDNA using Random Hexamers (Eurofins MWG Operon, Ebersberg, Germany) and the MMLV (Moloney Murine Leukemia Virus) Reverse Transcriptase (Takara Bio Inc., Shiga, Japan). RT-qPCR was performed via employment of qPCRBIO SyGreen Lo-ROX mix (PCR Biosystems, London, England, UK), in 8-tube strips and an MX300P Quantitative PCR System (Agilent-Stratagene, Santa Clara, CA, USA). For detection of gene expression, gene-specific primers were custom-designed (Table S1). Gene expression levels were normalized by *Importin 8* (*IPO8*) or *Glyceraldehyde-3-Phosphate Dehydrogenase* (*GAPDH*) mRNA respective ones. Relative gene expression was calculated using the 2^−ΔΔCt^ method [84]. Mean and standard deviation (SD) values were calculated, including 3 technical replicates, while biological experiments were performed at least 2 times.

### 4.7. Western Blotting

For protein expression analysis, cells were plated in 6 cm culture dishes, and incubated at +37 °C, and 5% CO_2_ (>95% humidity), until they reached ~90% confluency. At specific time points following treatment, and/or transfection/transduction, cells were collected by mild scraping and centrifuged at 2500× *g* for 5 min, and the obtained cell pellets were washed 3 times in 1× PBS. Following a second round of centrifugation at 2500× *g* for 5 min, cell pellets were resuspended in cold RIPA (Radio-Immuno-Precipitation Assay) Lysis Buffer (Pierce^TM^ RIPA buffer; Thermo Scientific, Waltham, MA, USA), containing Protease Inhibitors (Halt^TM^ Protease Inhibitor Cocktail; Thermo Scientific, Waltham, MA, USA) and 1 mM PMSF (Phenyl-Methyl-Sulfonyl-Fluoride) (Sigma-Aldrich, St. Louis, MO, USA) for 5 min, then centrifuged at 14,000× *g* for 15 min at +4 °C. Protein concentration was measured with BCA (Bi-Cinchoninic Acid, or Smith) Assay (BCA Assay Protein Kit; Cell Signaling Technology, Danvers, MA, USA). Cell lysates containing 6% SDS (Sodium-Dodecyl-Sulfate)-sample buffer were boiled for 10 min, and 25 μg of total protein from each cell lysate were subjected to SDS-Poly-Acrylamide Gel Electrophoresis (SDS-PAGE), then subsequently, transferred onto PVDF (Poly-Vinyli-Dene-Fluoride) Membrane(s) (Immobilon-P SQ Membrane, PVDF, 0.2 µm; Merck-Millipore, Burlington, MA, USA), using a Mini Trans-Blot Cell Transfer System (Bio-Rad, Hercules, CA, USA). The membranes were probed with the following Primary Antibodies: p21^CIP1/WAF1^ (Cell Signaling Technology, Danvers, MA, USA; #2947), p27^KIP1^ (Cell Signaling Technology, #9932), CDK2 (Cell Signaling Technology, #2546), CDK4 (Cell Signaling Technology, #12790), CDK6 (Cell Signaling Technology, #3136), Cyclin D3 (Cell Signaling Technology, #2936), p53 (Cell Signaling Technology, #9282), Phospho-p53 (Ser^15^) (Cell Signaling Technology, #9284), Caspase-3 (Cell Signaling Technology, #9662) Cleaved/Activated Caspase-3 (Cell Signaling Technology, #9664), Survivin (Cell Signaling Technology, #2808), LC3B(I/II) (Cell Signaling Technology, #43566), p62/SQSTM1 (Cell Signaling Technology, #5114), PARP1 (Cell Signaling Technology, #9532), BMAL1 (Cell Signaling Technology, #14020), REV-ERBα (Cell Signaling Technology, #13418), Phospho-Histone H2A.X (γH2AX) (Cell Signaling Technology, #9718), beta-Actin (*β*-Actin) (Origene Technologies, Rockville, MD, USA; #TA310155), and CRY2 (Origene Technologies, #TA502905S). After incubation with the respective HRP-conjugated Secondary Antibody (Anti-rabbit IgG, #7074; Anti-mouse IgG, #7076; Cell Signaling Technology), signals were detected using the Immobilon Classico Western HRP Substrate (Merck-Millipore, Burlington, MA, USA) and obtained images were acquired by the Fujifilm LAS-4000 Imaging System (Fujifilm Corporation, Tokyo, Japan). Data were analyzed by the Bio-Rad Image Lab Software, Version 6.1 (Bio-Rad, Hercules, CA, USA).

### 4.8. Plasmids

The EGFP-expressing shRNA constructs, targeting *BMAL1* (TL314656A, TL314656B, TL314656C, and TL314656D), and the Scramble control Vector (TR30021), were obtained from Origene Technologies (Rockville, MD, USA). These constructs were specifically used for the knock-down of BMAL1 expression. Scramble gRNA Lentiviral control Vector pLV[CRISPR]-hCas9/Puro-U6>Scramble_gRNA1 and Dual gRNA mammalian CRISPR Lentiviral Vector pLV [2CRISPR]-hCas9:T2A:Puro-U6>{hCRY2[gRNA#1]}-U6>{hCRY2[gRNA#2]}, targeting *CRY2*, were constructed and packaged by VectorBuilder (Chicago, IL, USA). These constructs were designed and engaged for the targeted disruption of *CRY2* gene, using the CRISPR/Cas9 technology. The Vector ID for Scramble is VB010000-9355sqw, and VB240129-1545dxv for the Dual gRNA CRISPR/hCas9 Vector (detailed information can be retrieved from “vectorbuilder.com”; accessed on 3 March 2024). The Lentivirus Packaging Plasmid psPAX2 (Addgene, Watertown, MA, USA; Plasmid #12260) and the Envelope Plasmid pMD2.G (Addgene, #12259) were kindly and generously provided by Professor Didier Trono (School of Life Sciences, École Polytechnique Fédérale de Lausanne, Lausanne, Switzerland). All Plasmids were verified with Restriction-site Digestion and Agarose-gel Electrophoresis.

### 4.9. Evaluation of BMAL1 Knock-Down Efficiency

To determine the most effective shRNA construct for *BMAL1* knock-down, HEK-293T cells were transfected with Scramble and 4 different shRNA plasmids (TL314656A, TL314656B, TL314656C, and TL314656D), using the Calcium Phosphate (Sigma-Aldrich, St. Louis, MO, USA) method [85]. Transfections were performed in ~50% confluent HEK-293T cells, seeded in 10 cm culture dishes. Transfection efficiency was assessed by monitoring GFP expression intensity using Fluorescence Microscopy at 24 and 48 h post-transfection. Subsequently, BMAL1 protein levels were evaluated via Western blotting, to quantify knock-down efficiency. Among the 4 constructs tested, the shRNA construct that exhibited the highest reduction in BMAL1 protein levels compared to the Scramble control was selected for subsequent Lentiviral production and knock-down experiments.

### 4.10. Lentivirus Production

For Lentivirus production, HEK-293T cells were seeded in a 10 cm culture dish until ~80% confluency. Two hours post-transfection, old medium was replaced with fresh complete medium lacking antibiotics. Cells were co-transfected with 4.2 μg psPAX2, 2.5 μg pMD2.G, and 5.8 μg *BMAL1* knock-down Plasmid (or Scramble control Plasmid), or 7.2 μg psPAX2, 4.0 μg pMD2.G, and 11.5 μg *CRY2* knock-out CRISPR/hCas9 Plasmid (or Scramble control Plasmid), using Calcium Phosphate protocols [85]. Conditioned supernatant, containing Lentivirus particles, was harvested, at 24 and 48 h post-transfection, then centrifuged at 4000× *g* for 15 min to remove cell debris. Pooled supernatants were filtered (0.45 μm Syringe Filter, Millex-HP; Merck-Millipore, Burlington, MA, USA) and used for Lentiviral transduction. Viral RNAs were extracted through employment of the NucleoSpin RNA Virus Kit (Macherey-Nagel, Düren, Germany) and titrated via engagement of the Lenti-X™ RT-qPCR Titration Kit (Takara Bio Inc., Shiga, Japan).

### 4.11. Lentivirus Transduction

AsPC-1 cells were transduced with 8 mL virus filtrate, including 8 μg/mL Polybrene (Santa Cruz Biotechnology, Dallas, TX, USA), in a 10 cm culture dish. Supernatant was replaced after 6 to 8 h with fresh complete medium (supplemented with 20% FBS). For *BMAL1* knock-down, transduction efficiency was quantified by GFP intensity, through Fluorescence Microscopy and polyclonal cell populations, expressing GFP, were further used for downstream experiments. For *CRY2* knock-out, stably transduced cells were clonally selected, using 2.5 μg/mL Puromycin Di-Hydro-Chloride (BioChemica, PanReac-Applichem, Darmstadt, Germany), for 1 week, and 5 μg/mL, for another 3 weeks.

### 4.12. Genomic DNA Extraction and PCR Amplification

For the validation and genotyping of CRISPR/hCas9-mediated knock-out cells, genomic DNA (gDNA) was extracted, using the Monarch Genomic DNA Purification Kit (New England Biolabs, Ipswich, MA, USA), according to manufacturer’s instructions. Genomic PCR amplification of the expected ~4.8 Kb gDNA fragment, encompassing the target sequence, was performed, using custom-designed primers (Table S1), and the Q5 High-Fidelity DNA Polymerase (New England Biolabs, Ipswich, MA, USA). Produced Amplicons were separated in (1%) agarose-gel electrophoresis.

### 4.13. Statistical Analysis

Experiments were performed at least 2 times, with at least 3 technical replicates each. IC_50_ values were calculated with the non-linear fit dose–response model. Data are provided as mean ± SD (Standard Deviation) or SEM (Standard Error of the Mean) values. *p*-value < 0.05 was considered as statistically significant. Significance was analyzed by unpaired Welch’s *t*-test and one-way or two-way ANOVA, with Tukey’s multiple comparisons test, unless otherwise stated, using the Prism Software, Version 10.3 (GraphPad Software, San Diego, CA, USA). All Western blotting experiments were performed at least 2 times, with densitometry plots corresponding to one representative Western blotting image.

## 5. Conclusions

Altogether, this study demonstrates the potential and promise of TH301, a novel CRY2 molecular stabilizer, to act as a new therapeutic agent for Pancreatic Ductal Adeno-Carcinoma (PDAC). TH301 effectively inhibits PDAC cell proliferation, induces cell cycle arrest at the G1-phase, promotes apoptosis, and activates autophagy via engagement of molecular mechanisms that function independently of the canonical p53 pathway. Interestingly, TH301 enhances the cytotoxic effects of Chloroquine and the conventional chemotherapeutic drug Oxaliplatin, thereby suggesting the need for prompt employment of TH301 in combination therapy schemes to overcome drug resistance often developed in PDAC and other aggressive malignancies. Most importantly, the p21 induction observed herein, in response to TH301 treatment, seems to occur independently of both CRY2 and BMAL1 modulation, thus indicating the involvement of alternative pathways, successfully operating in pancreatic cancer cell environments of diverse mutational loads.

Our findings place TH301 in a unique position, as a novel, powerful, and promising agent, for further development of new regimens dedicated to treatment of PDAC, and presumably other baneful cancers, whereat circadian clock disruption serves as a contributing factor to disease initiation, progression, metastasis, and/or chemoresistance. Future research must focus on elucidating the precise molecular mechanisms by which TH301 exerts its anti-cancer effects, and also on exploring its chemotherapeutic efficacies in vivo.

## Data Availability

All data are available in the Main Text or Supplementary Materials.

## Supplementary Materials

The following supporting information can be downloaded at: https://www.mdpi.com/article/10.3390/ijms26010178/s1.
