# Supplementary material for: TH301 Emerges as a Novel Anti-Oncogenic Agent for Human Pancreatic Cancer Cells: The Dispensable Roles of p53, CRY2 and BMAL1 in TH301-Induced CDKN1A/p21CIP1/WAF1 Upregulation
Source: Int J Mol Sci. 2024 Dec 28;26(1):178. doi: 10.3390/ijms26010178 (PMC11720130; doi:10.3390/ijms26010178)
Supplement: Supplementary file 1 [file ijms-26-00178-s001.zip › ijms-3376913-supplementary.pdf]

International Journal of
Molecular Sciences

MDPI

TH301 Emerges as a Novel Anti-oncogenic Agent for Human Pancreatic Cancer Cells: The Dispensable Roles of p53, CRY2 and BMAL1 in TH301-Induced *CDKN1A*/p21$^{CIP1/WAF1}$ Upregulation

Danae Farmakis [1,2], Dimitrios J. Stravopodis [1,*] and Anastasia Prombona [2]

[1] Section of Cell Biology and Biophysics, Department of Biology, School of Science, National and Kapodistrian University of Athens (NKUA), Panepistimiopolis, Zografou, 157 01 Athens, Attiki, Greece (GR); danaefar@bio.demokritos.gr (D.F.); dstravop@biol.uoa.gr (D.J.S.)

[2] Laboratory of Chronobiology, Institute of Biosciences and Applications (IBA), National Centre for Scientific Research (NCSR) "Demokritos", 153 41 Aghia Paraskevi, Attiki, Greece (GR); prombona@bio.demokritos.gr (A.P.)

*Correspondance: Dimitrios J. Stravopodis (D.J.S.), E-mail: dstravop@biol.uoa.gr

Abstract: *Background*: Pancreatic Ductal Adeno-Carcinoma (PDAC) is a highly aggressive cancer, with limited treatment options. Disruption of the circadian clock, which regulates key cellular processes, has been implicated in PDAC initiation and progression. Hence, targeting circadian clock components may offer new therapeutic opportunities for the disease. This study investigates the cytopathic effects of TH301, a novel CRY2 stabilizer, on PDAC cells, aiming to evaluate its potential as a novel therapeutic agent. *Methods*: PDAC cell lines (AsPC-1, BxPC-3 and PANC-1) were treated with TH301, and cell viability, cell cycle progression, apoptosis, autophagy, circadian gene and protein expression profiles were analyzed, using MTT assay, flow cytometry, Western blotting and RT-qPCR technologies. *Results*: TH301 proved to significantly decrease cell viability and to induce cell cycle arrest at the G1-phase across all PDAC cell lines, and especially the AsPC-1 and BxPC-3 ones, herein examined. It caused dose-dependent apoptosis and autophagy, and it synergized with Chloroquine and Oxaliplatin to enhance anti-oncogenicity. The remarkable induction of p21 by TH301 was shown to follow clock- and p53-independent patterns, thereby indicating the critical engagement of alternative mechanisms. *Conclusions*: TH301 demonstrates significant anti-cancer activities in PDAC cells, thus serving as a promising, new therapeutic agent, which can also synergize with approved treatment schemes, by targeting pathways beyond circadian clock regulation. Altogether, TH301 likely opens new therapeutic windows for the successful management of pancreatic cancer, in the clinical practice.

Keywords: apoptosis; autophagy; BMAL1; *CDKN1A*; circadian clock; CRY2; PDAC; p21$^{CIP1/WAF1}$; p53; TH301

SUPPLEMENTARY MATERIAL

Table S1. Oligo-nucleotide Primer Sequences

	RT-qPCR Primer Sequences	
Gene	**Forward (F)**	**Reverse (R)**
BMAL1	F: 5′-GCCCATTGAACATCACGAGTA-3′	R: 5′-CCTGAGCCTGGCCTGATAGTA-3′
NR1D1	F: 5′-GCAAGGGCTTTTTCCGTCG-3′	R: 5′-GCGGACGATGGAGCAATTCT-3′
NR1D2	F: 5′-GGGATGTGGCGTCAGGATTC-3′	R: 5′-TCAGGCACTTCTTGTACTGGATG-3′
PER2	F: 5′-ATTGTGAAGAATGCCGATATG-3′	R: 5′-CAACCTGGTGAGAGATGTAC-3′
CRY2	F: 5′-AGAACCACGACGAGACCTAC-3′	R: 5′-TTCCGTTCCAAGTGCTTATCC-3′
RORα	F: 5′-GAATCACCGAGAAGATGGAATAC-3′	R: 5′-AATGAAGTCGCACAATGTCTG-3′
RORγ	F: 5′-GGAAGTGGTGCTGGTTAGG-3′	R: 5′-GGGAGTGGGAGAAGTCAAAG-3′
TP53	F: 5′-GTTCCGAGAGCTGAATGAGG-3′	R: 5′-TTATGGCGGGAGGTAGACTG-3′
CDKN1A	F: 5′-CGCTCTACATCTTCTGCCTTAGTC-3′	R: 5′-GAACCTCTCATTCAACCGCCTAG-3′
NANOG	F: 5′-ATAGCAATGGTGTGACGCAGAAG-3′	R: 5′-CTCCAGGTTGAATTGTTCCAGGTC-3′
OCT4	F: 5′-TTCAGCCAAACGACCATCT-3′	R: 5′-TCGATACTGGTTCGCTTTCT-3′
SOX2	F: 5′-ATGGACAGTTACGCGCAC-3′	R: 5′-GCTGGTCATGGAGTTGTACTG-3′
IPO8	F: 5′-ACCGTTCCTCCTGAGAGACTCTG-3′	R: 5′-TGTGACATTTCCTGGGCTTCC-3′
GAPDH	F: 5′-CAATGACCCCTTCATTGAC-3′	R: 5′-GATGGTGATGGGATTTCC-3′
	PCR Primer Sequences for Gene Knock-out Validation	
CRY2	F: 5′-CACTTCACATAGCACAAC-3′	R: 5′-TTCTATTATCGTCATCATCTG-3′

Table S2. Oncogenic Alterations in (Human) PDAC Cell Lines. Data were Retrieved from the Dependency Map (DepMap) Portal (https://depmap.org)

PDAC CELL-LINE MUTATIONAL PROFILE

**GENE**	**AsPC-1**		**BxPC-3**		**PANC-1**	
	**Variant Type**	**Protein Change**	**Variant Type**	**Protein Change**	**Variant Type**	**Protein Change**
KRAS	SNV	p.G12D	WT	-	SNV	p.G12D
TP53	Deletion	p.C135AfsTer35	SNV	p.Y220C	SNV	p.R273H

CDKN2A	Deletion	p.L78HfsTer41	WT	-	WT	-
SMAD4	SNV	p.R100T	WT	-	WT	-
BRAF	WT	-	Deletion	p.V487_P492delinsA	WT	-

Abbreviations: SNV: Single Nucleotide Variant; WT: Wild-Type; fs: Frameshift; del: Deletion; ins: Insertion; Ter: Termination

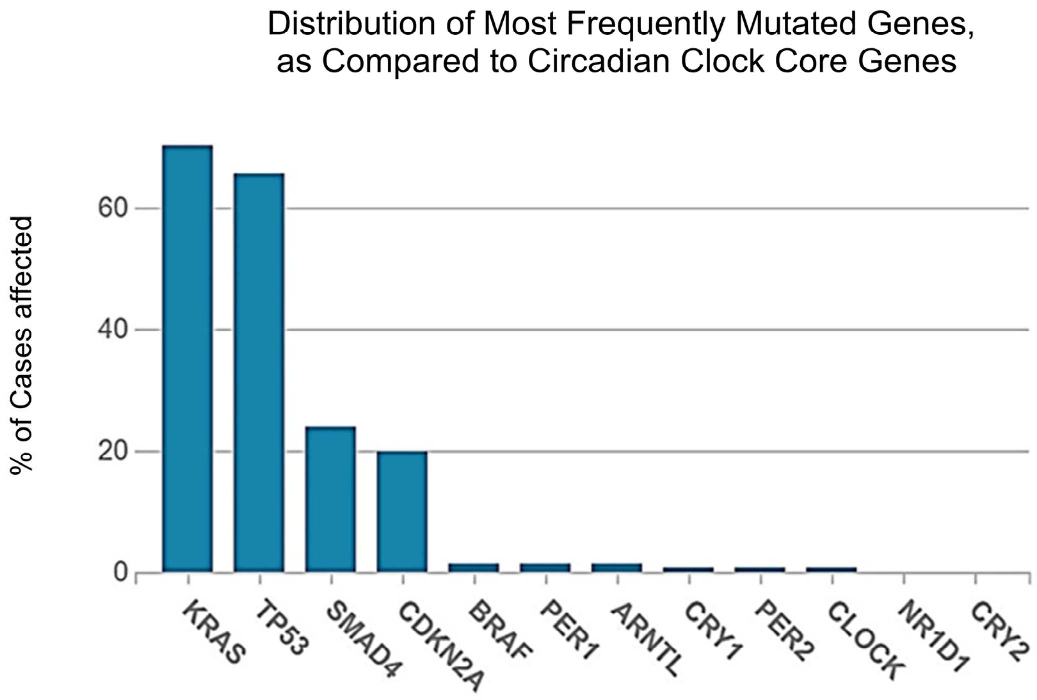

Figure S1. Mutational frequencies in the TCGA (The Cancer Genome Atlas) PAAD (PAncreatic ADenocarcinoma) patient population (185 cases). Bar-chart Plot retrieved from "The Cancer Genome Atlas" (https://portal.gdc.cancer.gov), after selecting for genes involved in pancreatic adenocarcinoma initiation and progression, and for core circadian clock genes, as well. Plot justifies low propability of mutations in core clock genes, especially *CRY2*.

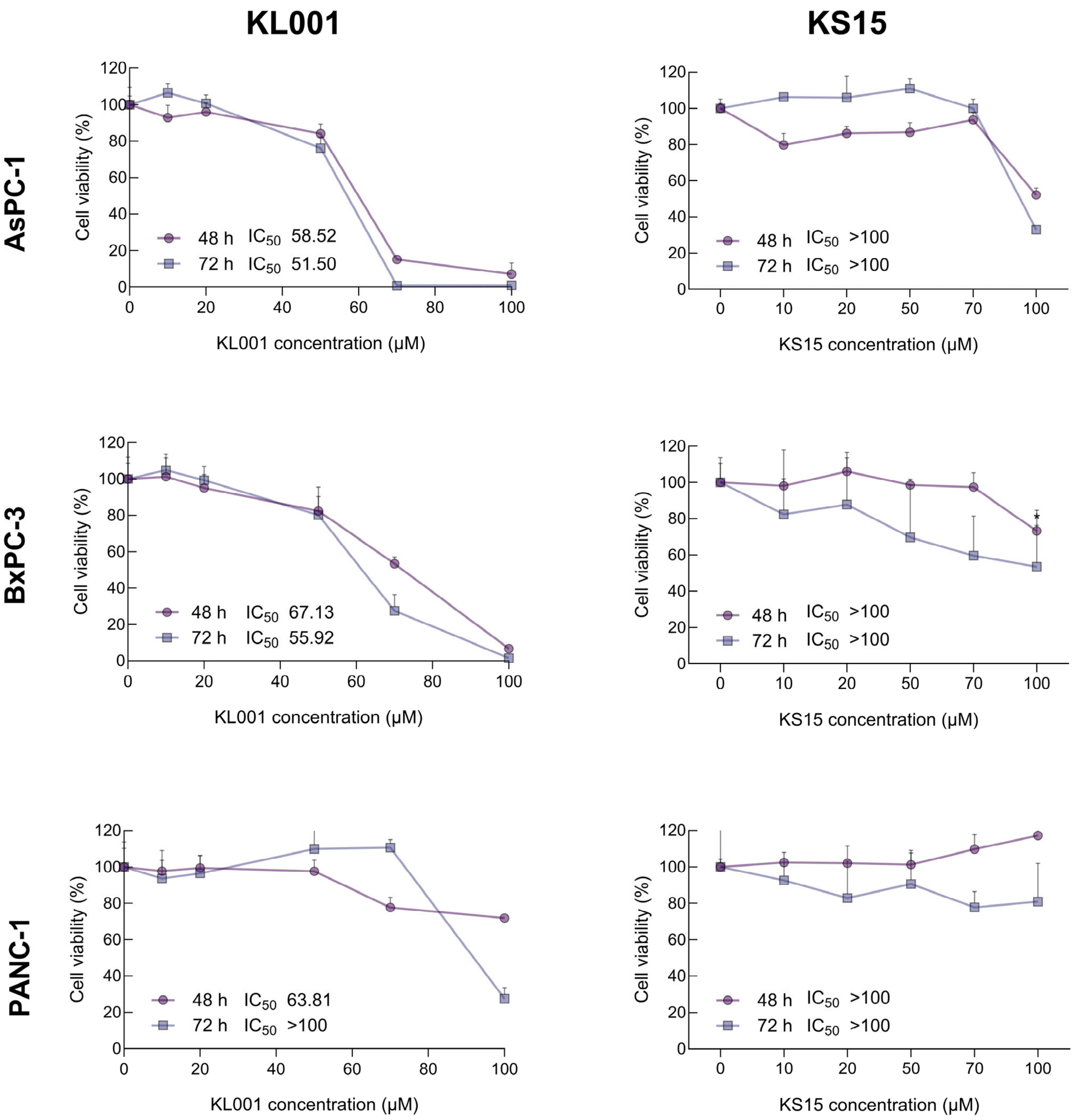

Figure S2. Effects of the CRY1/2 modulators KL001 and KS15 on PDAC cell viability. Percentage (%) of cell viability of PDAC cells after treatment with increasing concentrations of KL001 or KS15, for 48 and 72 h. Data (N = 3) are presented as Mean ± SD values. IC_{50} values were assessed with a non-linear regression model.

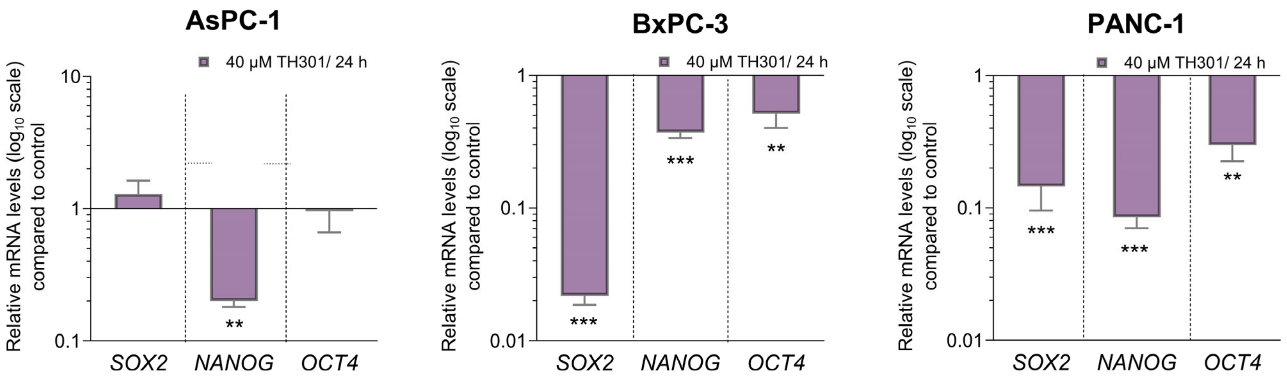

Figure S3. TH301 treatment reduces stemness-factor gene activities. Transcript mRNA levels of the stemness-factor genes *SOX2*, *NANOG* and *OCT4* in PDAC cells, following treatment with 40 μM TH301, for 24 h, being examined by RT-qPCR protocol engagement. mRNA values were normalized to the *IPO8* respective one, while control (0.1% DMSO) was set to value "1". Data (N = 3) are presented as Mean ± SD values. Statistical significance was assessed via Welch's t-test engagement. Asterisks indicate comparisons in between control (0.1% DMSO; not shown) and (TH301) treated cells, at significance levels of "0.05" and below values (*: < 0.05; **: < 0.01; ***: < 0.001).

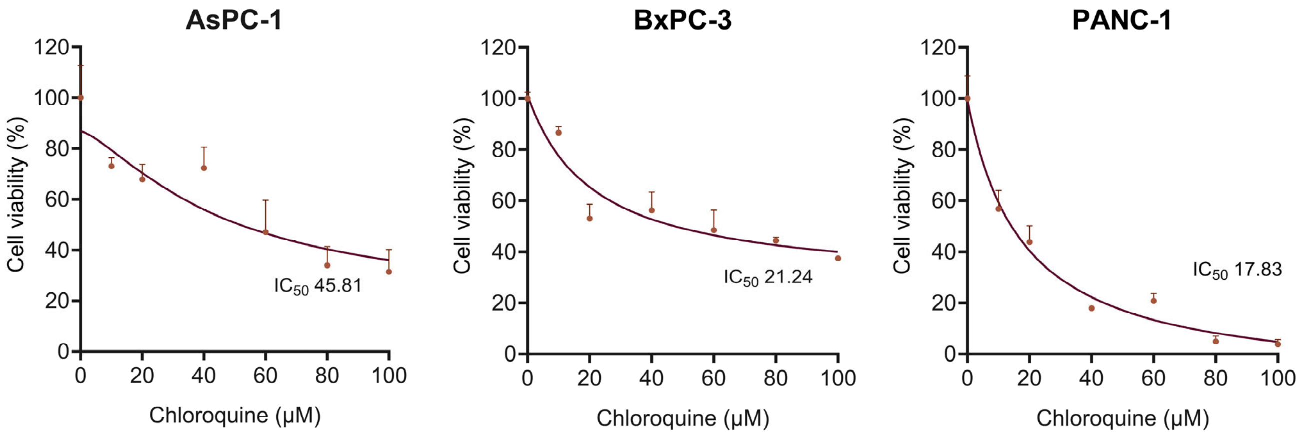

Figure S4. Effects of Chloroquine (CQ) on PDAC cell viability. Human pancreatic cancer cell lines were treated with increasing concentrations of CQ, for 48 h. Data (N = 4) are presented as Mean ± SD values. IC50 values were measured with a non-linear regression model.

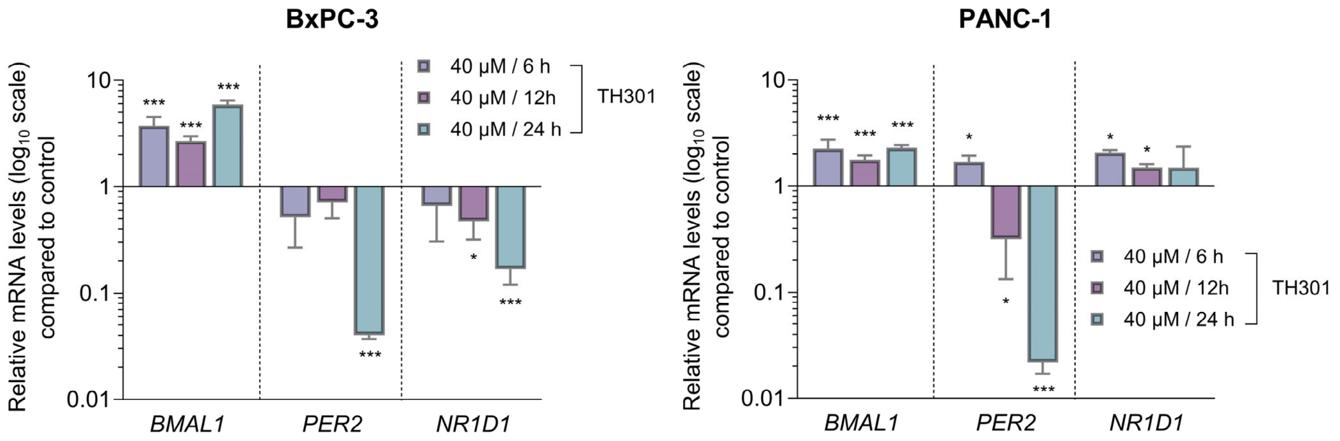

Figure S5. Effects of TH301 on circadian clock gene expression. Transcript mRNA levels of the circadian clock genes *BMAL1*, *PER2* and *NR1D1* in BxPC-3 and PANC-1 cells, following treatment with 40 µM TH301, for 6, 12 and 24 h, being determined by RT-qPCR protocol employment. mRNA values were normalized to the *IPO8* respective one, while control (0.1% DMSO) was set to value "1". Data (N = 3) are presented as Mean ± SD values. Statistical significance was assessed via Welch's t-test engagement, as each time-point was considered an independent experiment. Asterisks indicate comparisons in between control (0.1% DMSO; not shown) and (TH301) treated cells, at significance levels of "0.05" and below values (*: < 0.05; **: < 0.01; ***: < 0.001).

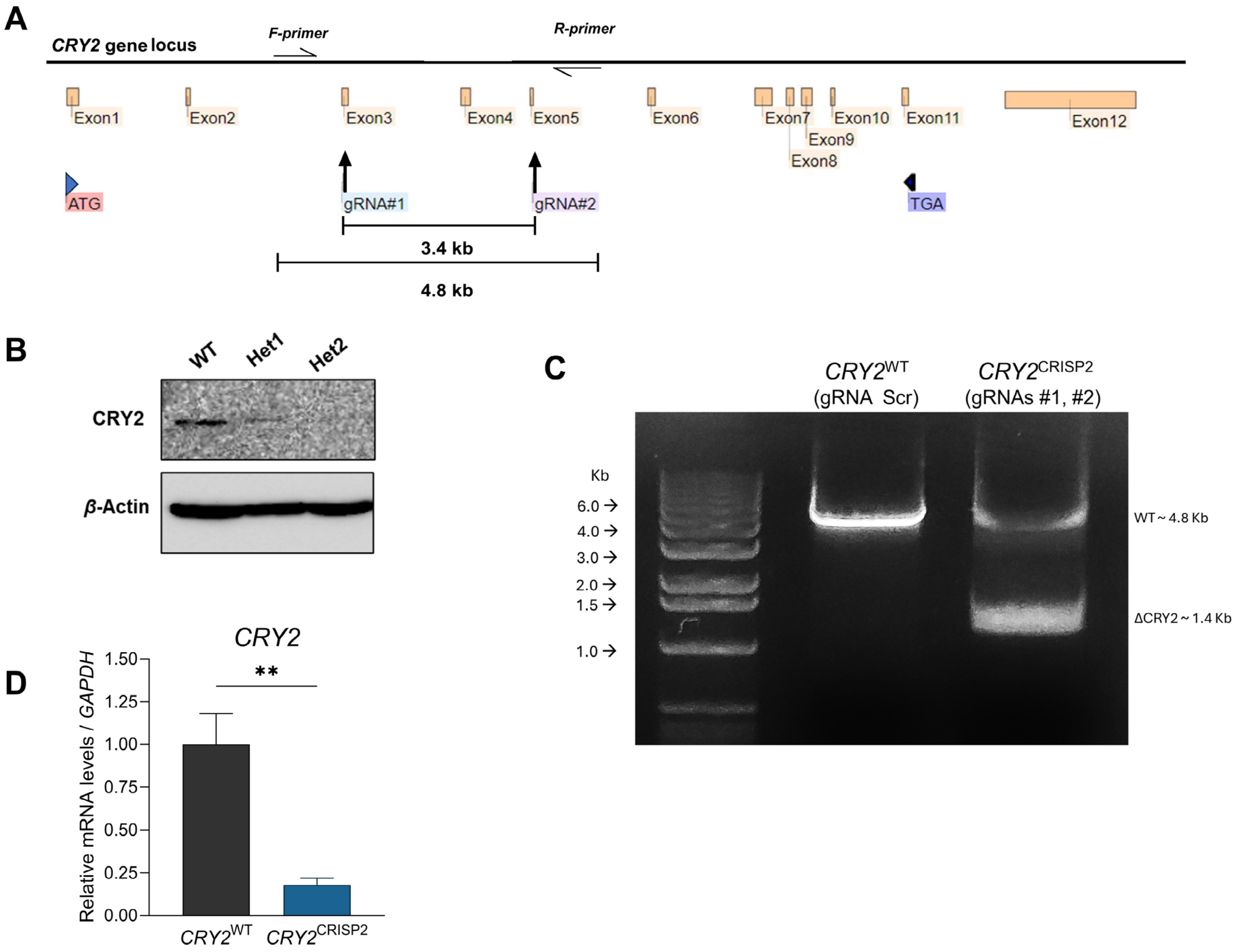

Figure S6. Molecular strategy and validation of CRY2-gene -partial- knock-out in AsPC-1 cells. A. CRISPR/hCas9-mediated CRY2-gene deletion design. The genomic region encompassing Exons 3-5 is approximately 4.8 Kb, in length, whereas, after the ~3.4 Kb deletion, the remaining region is reduced to ~1.4 Kb, in size. **B.** Western blotting, for the CRY2 protein expression comparison, in between wild-type (WT) and two different clones of $CRY2^{CRISP}$ AsPC-1 cells (Het1 and Het2). **C.** PCR genotyping, for the WT and $CRY2^{CRISP}$ (Het2: according to Western blotting results). In the genotyping of $CRY2^{CRISP}$, there are 2 distinct PCR products observed, thus indicating the partial knock-out of *CRY2* gene. **D.** Transcript mRNA levels of *CRY2* gene in WT and $CRY2^{CRISPR}$ (Het2) cells, being determined by RT-qPCR protocol engagement. mRNA values were normalized to the *GAPDH* one, while control (WT) was set to value "1". Data (N = 3) are presented as Mean ± SD values. Statistical significance was assessed with Welch's t-test employment. Asterisks indicate comparison in between WT and $CRY2^{CRISP}$ cells, at a significance level of "0.05" value (**: $p < 0.01$).

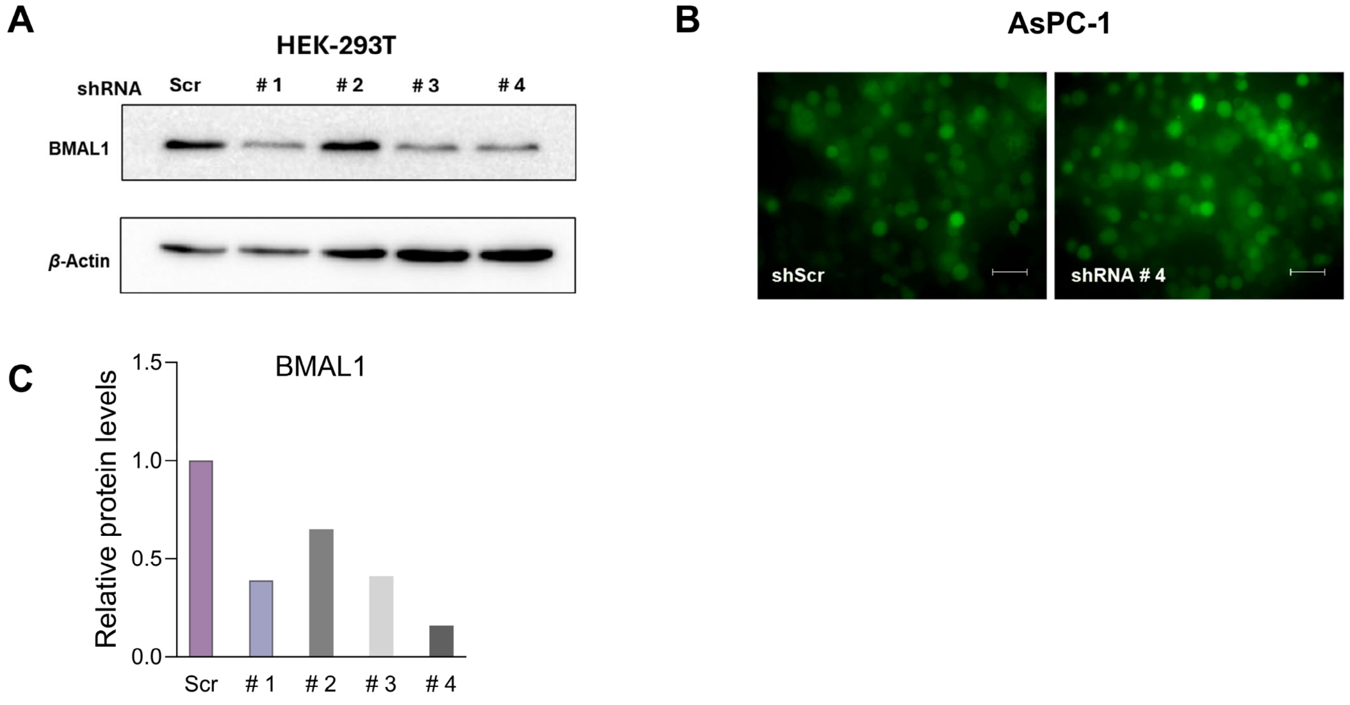

Figure S7. Validation of *BMAL1*-gene targeting efficacy. A. Western blotting, for screening of the BMAL1-gene silencing efficiency of 4 different (#1 - #4) shRNA species, in HEK-293T cells. Beta Actin (*β*-Actin) served as protein of reference (control). **B.** Transduction efficiency of Lentivirus shRNA #4 and Scramble (shScr) (control), in AsPC-1 cells (Scale bars 100 μm). **C.** Quantification of "A" conducted via measurements of densitometry values that were being normalized to *β*-Actin. Normalized values derived from Scramble (Scr) cells were set to "1".
